# Are global warming and ocean acidification conspiring against marine ectotherms? A meta-analysis of the respiratory effects of elevated temperature, high CO_2_ and their interaction

**DOI:** 10.1093/conphys/cow009

**Published:** 2016-03-23

**Authors:** Sjannie Lefevre

**Affiliations:** 1 Section for Physiology and Cell Biology, Department of Biosciences, University of Oslo, Oslo NO-0316, Norway

**Keywords:** Aerobic scope, climate change, fish, invertebrates, oxygen- and capacity-limited thermal tolerance, oxygen uptake

## Abstract

With the occurrence of global change, research aimed at estimating the performance of marine ectotherms in a warmer and acidified future has intensified. The concept of oxygen- and capacity-limited thermal tolerance, which is inspired by the Fry paradigm of a bell-shaped increase–optimum–decrease-type response of aerobic scope to increasing temperature, but also includes proposed negative and synergistic effects of elevated CO_2_ levels, has been suggested as a unifying framework. The objectives of this meta-analysis were to assess the following: (i) the generality of a bell-shaped relationship between absolute aerobic scope (AAS) and temperature; (ii) to what extent elevated CO_2_ affects resting oxygen uptake MO2rest and AAS; and (iii) whether there is an interaction between elevated temperature and CO_2_. The behavioural effects of CO_2_ are also briefly discussed. In 31 out of 73 data sets (both acutely exposed and acclimated), AAS increased and remained above 90% of the maximum, whereas a clear thermal optimum was observed in the remaining 42 data sets. Carbon dioxide caused a significant rise in MO2rest in only 18 out of 125 data sets, and a decrease in 25, whereas it caused a decrease in AAS in four out of 18 data sets and an increase in two. The analysis did not reveal clear evidence for an overall correlation with temperature, CO_2_ regime or duration of CO_2_ treatment. When CO_2_ had an effect, additive rather than synergistic interactions with temperature were most common and, interestingly, they even interacted antagonistically on MO2rest and AAS. The behavioural effects of CO_2_ could complicate experimental determination of respiratory performance. Overall, this meta-analysis reveals heterogeneity in the responses to elevated temperature and CO_2_ that is not in accordance with the idea of a single unifying principle and which cannot be ignored in attempts to model and predict the impacts of global warming and ocean acidification on marine ectotherms.

## Teaser

The changing climate prompts the need to study and ultimately predict the outcome for marine ectothermic animals. An analysis of the effects on respiratory performance of rising temperature, carbon dioxide, and their interaction, reveals diverse responses and a trend for additive and antagonistic rather than synergistic interactions.

## Introduction

**Figure 1:**
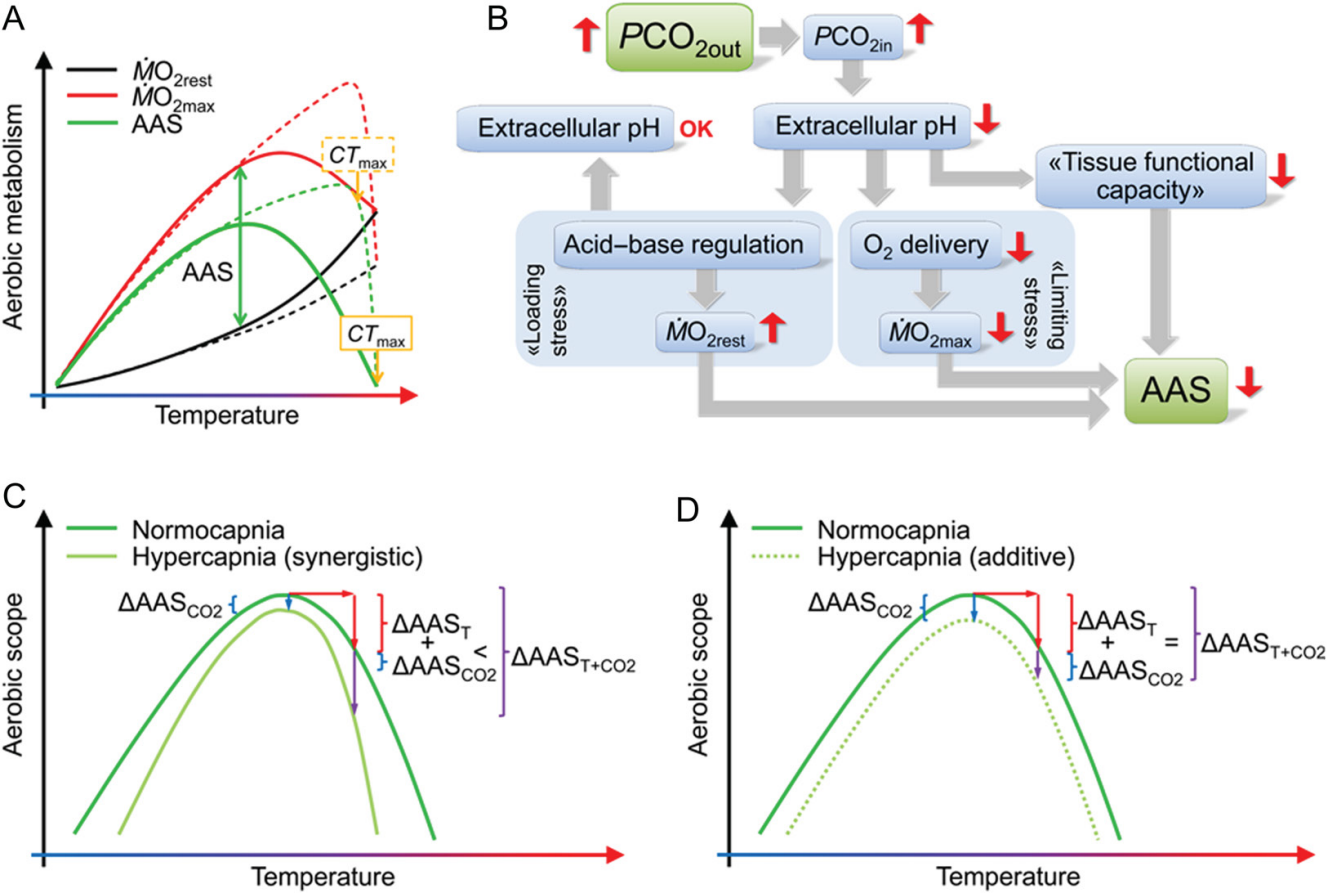
Proposed effects of temperature and CO_2_ on aerobic performance. (**A**) The Fry paradigm (continuous lines), where resting oxygen uptake ($\dot{M}O_{2\mathrm{rest}}$) is predicted to increase exponentially with temperature, whereas maximal oxygen uptake ($\dot{M}O_{2\max}$) reaches an optimum and then declines. As a result, the absolute aerobic scope ($\mathrm{ASS}=MO_{2\max}-MO_{2\mathrm{rest}}$) is represented by a bell-shaped curve, and the optimum for AAS (*T*_optAAS_) is assumed to reflect the overall optimum for fitness (*T*_optFIT_). Alternatively (dashed lines), it can be proposed that the increase in $\dot{M}O_{2\mathrm{rest}}$ may be less pronounced, because of acclimation, and that $\dot{M}O_{2\max}$ is not limited to the same degree at higher temperature (Clark *et al*., 2013; Schulte, 2015), resulting in a scope that continues to increase until the critical temperature *CT*_max_ (that is, where the animal eventually will die). In this case, it is unlikely that *T*_optAAS_ coincides with *T*_optFIT_. (**B**) Elevated CO_2_ in the water (*P*CO_2out_) has been proposed by some authors to cause a reduction in aerobic scope through ‘reduced tissue functional capacity’ (Pörtner, 2012) or by causing an elevation in $\dot{M}O_{2\mathrm{rest}}$ (loading stress) and/or a decrease in $\dot{M}O_{2\max}$ (limiting stress; Heuer and Grosell, 2014). (**C**) Change in the aerobic scope curve if simultaneous exposure to elevated temperature and hypercapnia has a synergistic effect, where the isolated effect of CO_2_ (ΔAAS_CO2_) at the thermal optimum is small, or zero, but exacerbated when temperature rises, causing further reduction in aerobic scope (e.g. Pörtner *et al*., 2005). The combined effect (ΔAAS*_T_*_+CO2_) is thus larger than expected from the sum of the two isolated effects (ΔAAS*_T_* and ΔAAS_CO2_). (**D**) Alternatively, hypercapnia can cause a reduction in AAS (ΔAAS_CO2_) independent of temperature, and the combined effect of the two stressors is then additive.

The influence of environmental temperature on the physiology of aquatic ectothermic animals has been extensively studied and, in most cases, the results are consistent with predictable effects of temperature on biological and chemical processes (e.g. Bělehrádek, 1930; Dell *et al.*, 2011). With the realization that Earth’s climate is changing—that global warming is happening—the focus on temperature in animal physiology research has obviously not subsided. One aspect that has received particular attention in this context is the capacity for aerobic metabolism, also referred to as scope for activity or simply aerobic scope. The absolute aerobic scope (AAS) is defined as the difference between the minimal and maximal rates of aerobic metabolism (Fry, 1971). Given that most processes occurring in an animal require ATP and that the most efficient ATP-producing pathway requires oxygen, AAS is regarded as an indicator of whole-animal performance that links directly to the capacity for activity, growth and reproduction and, thereby, ultimately fitness (Pörtner and Knust, 2007; Wang and Overgaard, 2007). Furthermore, the aerobic capacity is hypothesized to have an optimal temperature, below and above which AAS is reduced (Fry and Hart, 1948; Fry, 1971), and it follows then that the optimal temperature for AAS (*T*_optAAS_) will coincide with the optimum for other measures of performance and thereby overall fitness (*T*_optFIT_). According to this view, the decline in AAS that occurs above *T*_optAAS_ (Fig. 1A) is the result of an inability of the cardiorespiratory system to increase the maximal oxygen supply to tissues, $\dot{M}O_{2\max}$ (Pörtner and Farrell, 2008; Steinhausen *et al*., 2008; Munday *et al.*, 2012), at a rate that keeps pace with the expected exponential increase in basic oxygen demands (Clarke and Johnston, 1999) reflected by the resting oxygen uptake, $\dot{M}O_{2\mathrm{rest}}$ (Fig. 1A). This is a perfectly valid hypothesis that intuitively makes sense for many physiologists, while also being attractive from a modelling perspective (e.g. Farrell *et al.*, 2008, Del Raye and Weng, 2015). 

Although it is unlikely that Fry was concerned about the potential implications for global-warming research, his model did form the basis for a more recently developed framework that presents the Fry paradigm and aerobic-scope curve in a perspective more applicable to climate change, namely the concept of ‘oxygen- and capacity-limited thermal tolerance’ (OCLTT; e.g. Pörtner, 2001, 2010, 2014; Pörtner *et al.*, 2004a; Pörtner and Knust, 2007; Pörtner and Lannig, 2009; Bozinovic and Pörtner, 2015). This framework has, for example, formed the basis for conclusions drawn in the most recent report from the Intergovernmental Panel on Climate Change (IPCC), particularly regarding future biodiversity (Pörtner *et al.*, 2014). This framework has been presented as a ‘unifying concept’ (e.g. Farrell, 2016) and is used as an argument to correlate temperature-related shifts in distributions of animals with one specific variable, namely aerobic scope (Pörtner and Farrell, 2008). Indeed, AAS has, for example, been used to predict spawning success or to explain failure in migrating salmon populations (Farrell *et al.*, 2008; Eliason *et al.*, 2011), to compare habitat suitability between native and invasive fish species (Marras *et al*., 2015), to compare seasons (Cucco *et al*., 2012) and to predict the impact of climate change on the distribution and abundance of yellowfin tuna (Del Raye and Weng, 2015).

The generality of a compromised oxygen delivery that limits $\dot{M}O_{2\max}$ at higher temperatures has been questioned, because in some species $\dot{M}O_{2\max}$ and AAS continue to increase until temperatures close to the critical or incipient lethal limits, *CT*_max_ (Fig. 1A; Clark *et al*., 2013; Ern *et al*., 2014; Schulte, 2015). To be fair, Farrell (2009) did present a broader variety of AAS curves, and Farrell (2013) also argued that the ‘alternatively’ shaped curve was indeed recognized by Fry. This is true, as Fry (1947) in fact presented unpublished data from thermally acclimated brown bullhead catfish (*Ameiurus nebulosus*), where AAS clearly did not decline, even at 35°C, which is close to *CT*_max_ of that species. This recognition, however, is not apparent from the papers describing the OCLTT hypothesis. Furthermore, it can be questioned whether the underlying assumption that $\dot{M}O_{2\mathrm{rest}}$ increases exponentially is true for all species, when they are given sufficient time to acclimate (e.g. Sandblom *et al*., 2014). In any event, the result is an AAS curve that continues to increase until just before *CT*_max_. For OCLTT, *CT*_max_ is assumed to coincide with the point where AAS is zero, anaerobic metabolism is assumed to be invoked at this point (e.g. Frederich and Pörtner, 2000; Pörtner, 2001; Sokolova and Pörtner, 2003), and *CT*_max_ is thus determined entirely by the capacity for oxygen delivery (van Dijk *et al*., 1999). However, this view has been challenged (e.g. Ern *et al*., 2014, 2015; Wang *et al*., 2014; Verberk *et al*., 2016), and it has been suggested that *CT*_max_ may be limited by other processes, such as the loss of neurological function (Prosser and Nelson, 1981; Prosser, 1991; Ern *et al*., 2015). Nevertheless, an animal can survive at *CT*_max_ for only a limited amount of time. Therefore, if AAS continues to increase until very close to *CT*_max_, *T*_optAAS_ will be at the very boundary of the thermal window, at temperatures that might not even be within the ecologically relevant range of the animal, and it is likely that the performance of other processes (e.g. growth and reproduction), and thus fitness, have reached their optima at lower temperatures (Clark *et al*., 2013). Thus, *T*_optAAS_ is unlikely to coincide with *T*_optFIT_ and is therefore unlikely to guide the distribution of that species.

The Fry paradigm (Fry, 1971) considered temperature a controlling factor, hypoxia a limiting factor, and other factors, such as salinity, masking. The role of temperature and hypoxia in the OCLTT model is based on the Fry paradigm. Fry also considered hypercapnia a limiting factor, but only in special conditions and predominantly in combination with hypoxia. The OCLTT hypothesis, in contrast, incorporates CO_2_ more directly, as could be considered necessary from a future perspective involving both continued global warming and ocean acidification (IPCC, 2013). It has been predicted that elevated CO_2_ (and thereby reduced pH) will act as a limiting factor much in the same manner as hypoxia (e.g. Pörtner *et al.*, 2005; Pörtner and Farrell, 2008). Briefly, it is proposed that elevated external CO_2_ leads to internal accumulation of CO_2_, which in turn leads to a reduction in internal pH and reduced ‘tissue functional capacity’ (Pörtner, *et al.*, 2005; Pörtner, 2012; neither provides a more specific definition of the expression) and, lastly, a reduction in whole-animal AAS (Fig. 1B). Physiologically, it is difficult to understand why the relatively modest increase in the partial pressure of CO_2_ (*P*CO_2_) that comes with ocean acidification should have an impact on AAS of marine ectotherms. Many of these animals, particularly fish, have evolved mechanisms to maintain internal pH, despite an increase in external *P*CO_2_ and decrease in pH (e.g. Heisler, 1984; Claiborne *et al.*, 2002; Heuer and Grosell, 2014), at levels many times higher (e.g. Brauner and Baker, 2009) than what is projected for the future. It is not obvious why these modest changes should not be compensated for fully, even after prolonged exposure, because studies have shown acclimatory changes in, for example, mRNA expression of many of the channels and transporters involved in acid–base regulation (e.g. Deigweiher *et al.*, 2008; Heuer and Grosell, 2014). In contrast, it has been argued that the new steady-state levels of pH, *P*CO_2_ and HCO_3_^−^ resulting from the compensatory regulation may interfere with aerobic capacity (Pörtner *et al.*, 2004b), possibly by reducing $\dot{M}O_{2\max}$ (limiting stress; Fry, 1971; Farrell, 2016). Although it can be argued that any type of regulation will have an energetic cost (loading stress; Fry, 1971; Farrell, 2016), and hence potentially impair AAS, the question is whether the cost of compensating for the modest changes in external *P*CO_2_ are great enough to cause an energetic deficit. That is to say, why would it cost more to regulate internal pH in tomorrow’s pH 7.8 ocean compared with today’s pH 8.2 ocean? Nonetheless, in the early descriptions of the hypothesis (Pörtner *et al.*, 2005), it was proposed that CO_2_ would cause reductions in AAS at the thermal extremes, thereby narrowing the thermal tolerance window (Pörtner, *et al.*, 2005; Metzger *et al*., 2007; Wittmann and Pörtner, 2013). In other words, it was suggested that elevated temperature and elevated CO_2_ would act synergistically (Fig. 1C). The possibility that a reduction in AAS is induced by CO_2_ itself has also been included (Pörtner and Farrell, 2008; Pörtner, *et al.*, 2014), in which case the effect of temperature and CO_2_ would simply be additive (Fig. 1D).

Several reviews have been written on the subject of ocean acidification and its effect on animal physiology and/or behaviour, either alone (e.g. Heuer and Grosell, 2014; Clements and Hunt, 2015; Nikinmaa and Anttila, 2015) or in combination with elevated temperature (e.g. Hofmann and Todgham, 2010; Harvey *et al.*, 2013; Parker *et al.*, 2013; Wittmann and Pörtner, 2013; Przeslawski *et al.*, 2015), but none of these included, in detail, $\dot{M}O_{2\mathrm{rest}}$ or AAS. With regard to temperature and the shape of the aerobic performance curve, Clark *et al*. (2013), Schulte (2015) and Farrell (2016) presented excellent discussions of the subject, but with focus on a few examples, and not a quantitative assessment of the available data. Likewise, a recent review by Verberk *et al*. (2016) limited their discussion of OCLTT to arthropods.

The objective of the present review is therefore to evaluate quantitatively the current knowledge on the effects of temperature, CO_2_ and their possible interaction on $\dot{M}O_{2\mathrm{rest}}$ and AAS, to see how well the data fit the predictions described above. Specifically, the following questions are asked. Does AAS in general follow a bell-shaped curve, i.e. is there always an optimal temperature? Does CO_2_ in general cause an increase in $\dot{M}O_{2\mathrm{rest}}$, and thereby, reduce AAS? And is the combined effect of CO_2_ and temperature on $\dot{M}O_{2\mathrm{rest}}$ and AAS generally larger than expected from their sum, i.e. is the interaction synergistic? In addition, the behavioural alterations caused by CO_2_, and implications thereof, are briefly discussed. It is important to clarify that this review is not about proving or disproving the OCLTT hypothesis *per se*, because that would require more studies with a mechanistic approach than what is currently available, but rather put it into the perspective of the data that exist and discuss the implications for future research on how global change may affect animal physiology.

## General approach

To answer the questions outlined above, a meta-analysis approach was adopted, similar to analyses by Harvey *et al*. (2013), Przeslawski *et al*. (2015) and Steckbauer *et al.* (2015). The log response ratio (lnRR) was chosen because it is intuitive, while also ensuring that effect sizes for different data sets are spread more evenly along a scale, making it easier to visualize graphically. A meta-analysis in the strictest sense has as output a single mean effect size for a given variable (Harvey *et al*., 2013), or even combining several variables (Przeslawski *et al*., 2015). This mean effect size can then be tested statistically against a hypothetical value (commonly zero), or between different animal groups and life stages. Although this approach is attractive because it gives straightforward ‘yes or no’ answers, it also introduces the risk that potentially interesting patterns and range of responses are overlooked. Given that many species are studied (unlike the situation in medical meta-analyses), the breadth of responses may reflect biological diversity. I therefore chose to examine the diversity of responses by presenting data graphically, rather than focusing only on mean effect sizes.

Three categories of studies were considered for the analyses. To be included, studies had to present data on one or more of the following three overall categories.

*Category A*. The AAS, calculated as the difference between $\dot{M}O_{2\mathrm{rest}}$ (fasted animals showing minimal activity) and $\dot{M}O_{2\max}$ (during swimming or another form of maximal activity, or after being chased or stressed to exhaustion), measured using respirometry, at three or more temperatures. In total, 87 data sets (from 48 papers on 53 species) were included in this category.
*Category B*. The $\dot{M}O_{2\mathrm{rest}}$ or AAS at two or more CO_2_ levels (one control and a minimum of one elevated). In total, 206 data sets on $\dot{M}O_{2\mathrm{rest}}$ (from 78 papers on 83 species) and 20 data sets on AAS (from 14 papers on 16 species) were included in this category.
*Category C*. The $\dot{M}O_{2\mathrm{rest}}$ or AAS at two or more CO_2_ levels (one control and a minimum of one elevated) in combination with two or more temperatures. In total, 70 data sets on $\dot{M}O_{2\mathrm{rest}}$ (from 32 papers on 43 species) and 12 data sets on AAS (from seven papers on eight species) were included in this category.

For studies reporting effects of three (or more) temperatures in combination with CO_2_, or vice versa, lnRR was calculated for each control–treatment contrast. As the primary goal of the analysis was not to calculate a mean effect size across studies, but also to explore the diversity and potential causes of variation, this method was chosen over calculation of an aggregate within-study lnRR (Lajeunesse, 2011). The goal was to include studies on both fish and invertebrates, irrespective of time frame, temperature ranges and CO_2_ regimes, but only if sample size and some form of variance estimate for each experimental group were reported or could be extracted from figures. The literature on fish, however, turned out to be dominated by studies on teleosts, whereas the literature on invertebrates was dominated by molluscs (gastropods, cephalopods and bivalves), echinoderms, cnidarians and crustaceans. These marine invertebrates are all calcifying to some extent. Data were extracted from papers using WebPlotDigitizer 3.8 (http://arohatgi.info/WebPlotDigitizer/citation.html). Details of each study, raw values ($\dot{M}O_{2\mathrm{rest}}$, $\dot{M}O_{2\max}$ and AAS for category A, or $\dot{M}O_{2\mathrm{rest}}$ and AAS for categories B and C), calculated lnRR and reference details, are collected in a Microsoft Excel spread sheet made available as online Supplementary Material. Data were plotted and analysed in GraphPad Prism 6.07 (GraphPad Software Inc., www.graphpad.com).

## Calculations

### Comparison of Fry aerobic-scope curves (category A)

Species differ in their AAS and hence the magnitude of change with temperature, and it was therefore necessary to express the changes with temperature in relative terms to be able to compare the shape of the curve between different species. The relative aerobic scope (RAS), relative resting oxygen uptake ($R\dot{M}O_{2\mathrm{rest}}$) and relative maximal oxygen uptake ($R\dot{M}O_{2\max}$), at different measurement temperatures (*T_i_*) within each data set, were therefore calculated as a percentage of the maximum:

(1) $$RX=\frac{X_{T_{i}}}{X_{\max}}\times 100,$$

where $X_{T_{i}}$ is AAS, $\dot{M}O_{2\mathrm{rest}}$ or $\dot{M}O_{2\max}$ at *T_i_*, and *X*_max_ is the highest value of AAS, $\dot{M}O_{2\mathrm{rest}}$ or $\dot{M}O_{2\max}$ within each data set. A similar approach has previously been adopted by other authors (Brett, 1971; Tirsgaard *et al*., 2015a). Furthermore, the temperature coefficient (*Q*_10_) for $\dot{M}O_{2\mathrm{rest}}$ was calculated for each data set according to equation 2:

(2) $$Q_{10}={\left(\frac{\dot{M}O_{2\mathrm{rest},2}}{\dot{M}O_{2\mathrm{rest},1}}\right)}^{10/(T_{2}-T_{1})},$$

where $\dot{M}O_{2\mathrm{rest},1}$ is the resting oxygen uptake at the lowest temperature, *T*_1_, whereas $\dot{M}O_{2\mathrm{rest},2}$ is the resting oxygen uptake at the temperature where it was highest, *T*_2_, thus excluding the highest temperatures if $\dot{M}O_{2}$ was reduced.

In all cases, the raw values from each study were used for calculations, although data were also converted to a standardized unit (milligrams of O_2_ per kilogram per hour) and body mass (100 g). The raw as well as standardized values ($\dot{M}O_{2\mathrm{rest}}$ and $\dot{M}O_{2\max}$) and calculated values (AAS, RAS, $R\dot{M}O_{2\mathrm{rest}}$ and $R\dot{M}O_{2\max}$) are shown in Supplementary Table ST1.

It was then determined for each data set whether a thermal optimum for aerobic scope (*T*_optAAS_) could be identified clearly or not. To use an objective criterion, *T*_optAAS_ was considered to be absent in a data set if RAS did not fall below 90%, to take variation and measurement error into account. To examine the effect of acclimation on the outcome, data were first divided into acute studies (0–5.5 days) and acclimation studies (7–365 days), and a two-by-two contingency table of the outcome (‘*T*_optAAS_ yes’ vs. ‘*T*_optAAS_ no’) and duration (acute vs. acclimation) was analysed by Fisher’s exact test. Additionally, to examine whether the outcome was significantly influenced by the methodology used to estimate $\dot{M}O_{2\max}$, a two-by-two contingency table of outcome (‘*T*_opt_ yes’ vs. ‘*T*_opt_ no’) and methodology ($\dot{M}O_{2\max}$ measured after chasing vs. $\dot{M}O_{2\max}$ measured during maximal activity) was analysed by Fisher’s exact test. To avoid bias, more than one data set on the same species from the same research group was included only if it differed in outcome or methodology, and the number of data sets used in these analyses (73) was therefore lower than the total number of data sets (87).

To examine whether the rate at which $\dot{M}O_{2\mathrm{rest}}$ increased with temperature affected the presence or absence of *T*_optAAS_, a Mann–Whitney *U*-test was used to determine whether the mean *Q*_10_ of data sets with different outcome (‘*T*_opt_ yes’ vs. ‘*T*_opt_ no’) and group (acute vs. acclimation) was significantly different. Given that raw data for $\dot{M}O_{2\mathrm{rest}}$ and $\dot{M}O_{2\max}$ were not presented for three species [hapuku wreckfish (*Polyprion oxygeneios*), salema (*Sarpa salpa*) and marbled spinefoot (*Signaus rivulatus*)], 84 data sets out of 87 were included in this analysis. Additionally, a Mann–Whitney *U*-test was used to determine whether the mean duration of studies with different outcome (‘*T*_opt_ yes’ vs. ‘*T*_opt_ no’) and group (acute vs. acclimation) was significantly different. Given that acclimation time was not specified in one case (brown bullhead), 86 data sets out of 87 were included in this analysis.

### Isolated effects of elevated CO_2_ on resting oxygen uptake and absolute aerobic scope (category B)

For each dataset, the effect size was calculated as the log response ratio, lnRR, between the measured value (*Y*) of a given variable ($\dot{M}O_{2\mathrm{rest}}$ or AAS) in a control condition, *Y*_ctrl_, and an experimental condition, *Y*_exp_, as described by equation 3 (Hedges *et al.*, 1999; Lajeunesse, 2011):

(3) $$\ln\mathrm{RR}=\ln\left(\frac{Y_{\exp}}{Y_{\mathrm{ctrl}}}\right).$$

For each effect size, where SD is the standard deviation and *n* is the sample size from each group, the variation of lnRR, *v*(lnRR), was calculated according to Hedges *et al.* (1999):

(4) $$v(lnRR)=\frac{{\left(SD_{\exp}\right)}^{2}}{n_{\exp}\times {\left(Y_{\exp}\right)}^{2}}+\frac{{\left(SD_{\mathrm{ctrl}}\right)}^{2}}{n_{\mathrm{ctrl}}\times {\left(Y_{\mathrm{ctrl}}\right)}^{2}}.$$

The 95% confidence interval (CI) was estimated based on *v*(lnRR) as suggested by Hedges *et al.* (1999):

(5) $$\begin{array}{l} \ln\mathrm{RR}-1.96\times \sqrt{v(lnRR)}\leq \ln\mathrm{RR}\geq \ln\mathrm{RR}\\ \;\;\;\;\;\;\;\;\;\;\;+1.96\times \sqrt{v(\ln\mathrm{RR})}. \end{array}$$

Given that there were both positive and negative values for lnRR, it was decided to present all values graphically, as opposed to presenting only a (weighted) mean effect size. Data were divided into different groups of invertebrates (bivalves, cephalopods, cnidarians, crustaceans, echinoderms and gastropods) and fish (teleosts and elasmobranchs) and plotted separately as a function of life stage (adult and non-adult), temperature, *P*CO_2_ in the experimental treatment and length of CO_2_ treatment, to investigate the degree of correlation (assessed by linear regression and Pearson’s *r*) between lnRR and these variables. Data points were also given different symbols to indicate taxonomic class, and different colours to indicate life stages (adult and non-adult). The data sets are available as Supplementary Tables ST2_MO2rest and ST2_AAS.

To examine whether methodology had a significant impact on the outcome, studies on $\dot{M}O_{2\mathrm{rest}}$ were divided into those where $\dot{M}O_{2\mathrm{rest}}$ was measured during a truly resting state (‘resting’) and those where some routine activity could not be ruled out (‘routine’), whereas studies on AAS were divided into those where $\dot{M}O_{2\max}$ was estimated immediately after exhaustive exercise (‘post-chase’) and those where it was measured during maximal activity (‘during exercise’). The lnRR values in different data sets were categorized as ‘no effect’ if the 95% CI overlapped zero and as ‘effect’ if the 95% CI did not overlap zero. Data sets in the latter group were then categorized further as ‘decrease’ if the lnRR was negative and ‘increase’ if lnRR was positive. More than one data set from the same research group on the same species was included only if it differed in outcome or methodology; therefore, 125 out of 206 data sets on $\dot{M}O_{2\mathrm{rest}}$ and 18 out of 20 data sets on AAS were included in these analyses. The resulting two-by-two contingency tables of the outcomes (effect vs. no effect, and decrease vs. increase) were then analysed using Fisher’s exact test.

### Combined effects of elevated temperature and CO_2_ (category C)

For both $\dot{M}O_{2\mathrm{rest}}$ and AAS in each data set, lnRR was calculated for each of the isolated effects of temperature and CO_2_ (lnRR*_T_* and lnRR_CO2_, respectively), as well as the combined effect (lnRR*_T_*_+CO2_), as described in equations 3, 4 and 5. To examine the nature of the interaction between temperature and CO_2_, the expected combined effect size for each data set, assuming a simple additive effect (lnRR_add_), was calculated as described by equation 6:

(6) $$\begin{array}{l} \ln RR_{\mathrm{add}}=\ln\left(\frac{Y_{\mathrm{add}}}{Y_{\mathrm{ctrl}}}\right)=\ln\left(\frac{Y_{\mathrm{ctrl}}+(Y_{\mathrm{temp}}-Y_{\mathrm{ctrl}})+(Y_{\mathrm{CO}2}-Y_{\mathrm{ctrl}})}{Y_{\mathrm{ctrl}}}\right)\\ \;\;\;\;\;\;\;\;\;\;\;\;\;\;\;\;\;\;\;=\ln\left(\frac{Y_{\mathrm{temp}}+Y_{\mathrm{CO}2}-Y_{\mathrm{ctrl}}}{Y_{\mathrm{ctrl}}}\right). \end{array}$$

More specifically, the additive effect was interpreted as the sum of the control value (*Y*_ctrl_), the change caused by temperature (*Y*_temp_ − *Y*_ctrl_) and the change caused by elevated CO_2_ (*Y*_CO2_ − *Y*_ctrl_). The observed lnRR*_T_*_+CO2_ for $\dot{M}O_{2\mathrm{rest}}$ and AAS in each data set could then be plotted as a function of the expected lnRR to visualize the range and direction of effects. From these plots, the presence of a synergistic, additive or antagonistic interaction could be determined for each point (i.e. study or data set), and also from the slope of the line fitted by linear regression (a similar approach was used by Steckbauer *et al.*, 2015). A slope of 1.0 would indicate perfect additivity; a slope <1 would indicate an antagonistic effect, and a slope >1 would indicate synergism. Data from invertebrates and fish were plotted separately, and data points were given different symbols to indicate taxonomic group and different colours to indicate life stages (adult and non-adult). The data sets are available as Supplementary Tables ST3_MO2rest and ST3_AAS.

## Results

### Aerobic-scope curves

**Figure 2:**
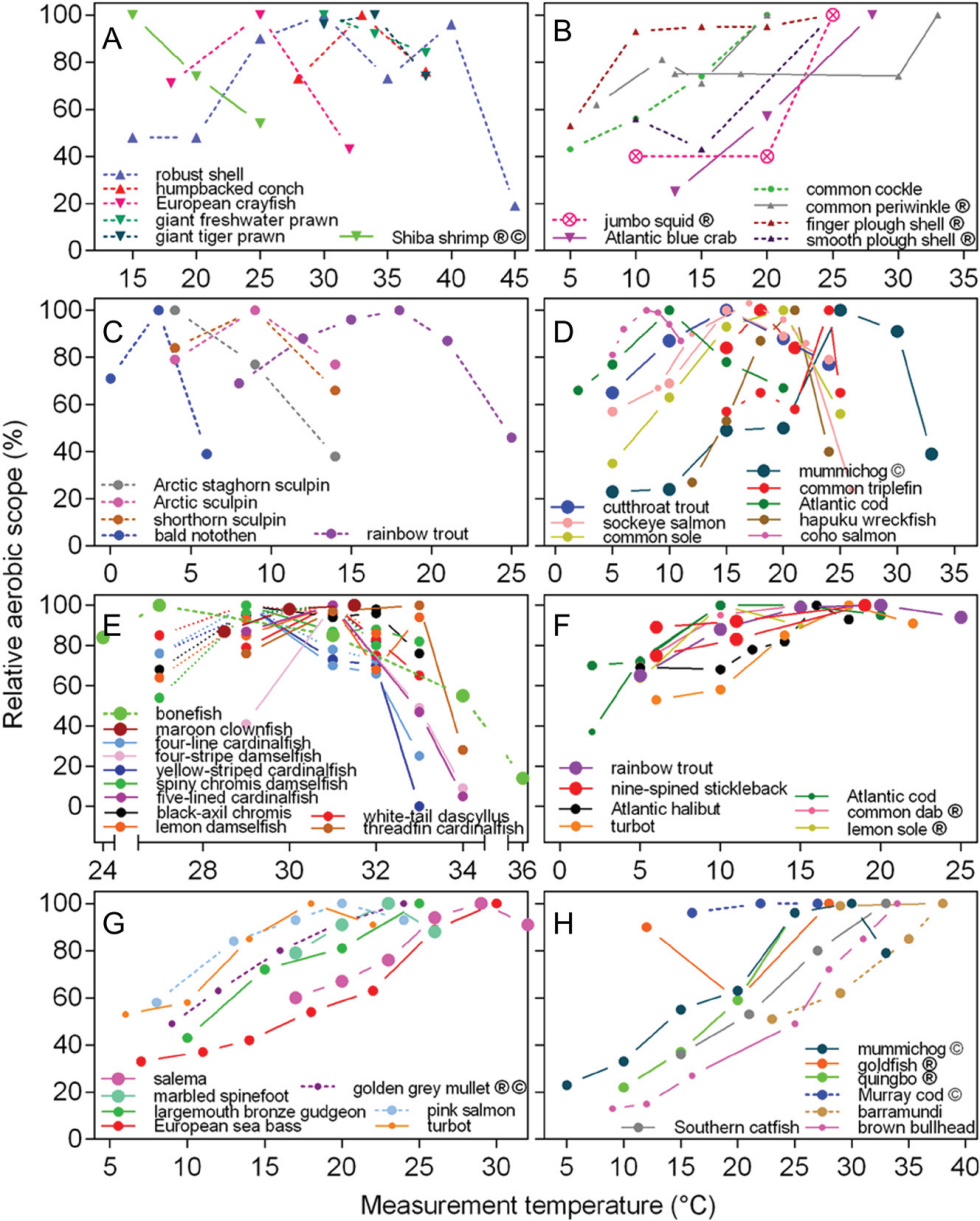
Relative aerobic scope as a function of temperature in different invertebrates and teleost fish. Aerobic scope was set to 100% at the temperature where it was highest. Only studies where measurements of absolute aerobic scope (AAS, measured as the difference between maximal and minimal oxygen uptake) were conducted at three or more temperatures are included. Data were not available for marine invertebrate groups other than gastropods (upright triangles), crustaceans (inverted triangles), bivalves (hexagons) and cephalopods (circled crosses) and for fish other than teleosts (circles). Continuous lines represent acclimated individuals (minimum of 7 days at each temperature), whereas dashed lines represents acute measurements (from 30 min to 5.5 days). Multiple lines of the same colours represent different studies on the same species or studies on different populations of the same species. Symbol size reflects sample size at each temperature (<5 = small, 5–10 = medium and >10 = large). For most species, the temperature range over which aerobic scope was measured is representative for the temperatures at which the species occurs, or even up to critical temperatures. Species for which the relevance of the tested temperature range is unclear are indicated by ‘•’. Species, where routine rather than resting oxygen uptake was measured are indicated by ‘©’. (**A**) Invertebrate species that appear to have an optimal temperature for AAS (*T*_optAAS_). (**B**) Invertebrate species where AAS increases over the entire investigated temperature range. However, in only two cases (Atlantic blue crab and common cockle) was the highest temperature also used close to the upper limit of the species. (**C**) Studies on cold-water or temperate fish species acutely exposed to elevated temperature. All have a clear *T*_optAAS_. (**D**) Studies on primarily temperate fishes after acclimation at different temperatures, where *T*_optAAS_ is evident. (**E**) Coral reef fishes that have a much narrower thermal range than the fishes in (D), but also showing declines in AAS with temperature (one exception being the maroon clownfish) and thus clear *T*_optAAS_. Also, there was an intraspecific effect of latitude, with warmer populations (continuous lines; Lizard Island and Papua New Guinea) showing a rightward shift in their thermal range and a greater decline in aerobic scope with temperature compared with the less warm populations (dotted lines; Heron Island). (**F**) Studies on temperate fish species after acclimation where *T*_optAAS_ is not clearly evident. (**G**) Studies on fish species with a slightly wider temperature range than species in (F), where *T*_optAAS_ is not clearly evident. (**H**) Freshwater fishes where a *T*_optAAS_ is not evident, except for mummichog (relative aerobic scope only declines from 100 to 75% at the highest temperature, which is the highest at which fish could be kept without mortality). The complete data set and reference details are available in Supplementary Table ST1. Note that several independent (but very similar) data sets exist for the coral reef fishes, but to enhance visual clarity of the graph only data from Rummer *et al*. (2014) and Gardiner *et al*. (2010) are shown.

The changes in relative aerobic scope (RAS) with temperature across different gastropod, bivalve and crustacean invertebrates are shown in Fig. 2A and B, whereas the changes in RAS with temperature across different teleost fish species are shown in Fig. 2C–H. 

The first group of invertebrates (Fig. 2A) all showed a clear *T*_optAAS_, even when only three temperatures were investigated. It is noteworthy that only one of these studies used thermally acclimated individuals (Shiba shrimp, *Metapenaeus joyneri*). The second group (Fig. 2B), in contrast, consists of invertebrates where a clear *T*_optAAS_ was not identified within the temperature range measured. In these cases, all but two [Atlantic blue crab (*Callinectes sapidus*) and common periwinkle (*Littorina littorea*)] were acute studies, and most studies [except common cockle (*Cerastoderma edule*) and Atlantic blue crab] used a temperature range that might not have reflected the tolerance of the species.

The teleost fish were divided into six panels according to similarities in duration, temperature range or response curves. Two main patterns emerged: species where a *T*_optAAS_ was clearly evident (Fig. 2C–E) and species where RAS did not decrease or only slightly so (remaining above 90% in most cases) at the highest temperature (Fig. 2F–H). These two patterns were present across different types of fishes (cold-water, Fig. 2C, D and F; warm-water, Fig. 2E, G and H; and freshwater, Fig. 2H) and different time frames (acute, Fig. 2C, G and H; and acclimated, Fig. 2D–H). Some species showed a clear *T*_optAAS_ over a broad temperature range (Δ*T* = 10–20°C; Fig. 2C and D), whereas others, particularly the coral reef fish and the bald notothen (*Pagothenia borchgrevinki*), did so over a narrower temperature range (Fig. 2C and E; Δ*T* = 4–7°C). For the coral reef fish, it was evident that the temperature window of a given species was not fixed, because it was right shifted in populations from warmer areas. Most of the studies where only minor decreases in RAS were observed were carried out on thermally acclimated individuals and covered the relevant temperature range for each species, such that the highest temperature used was slightly below the temperature where mortality occurred or the maximal temperature found in the habitat.

**Figure 3:**
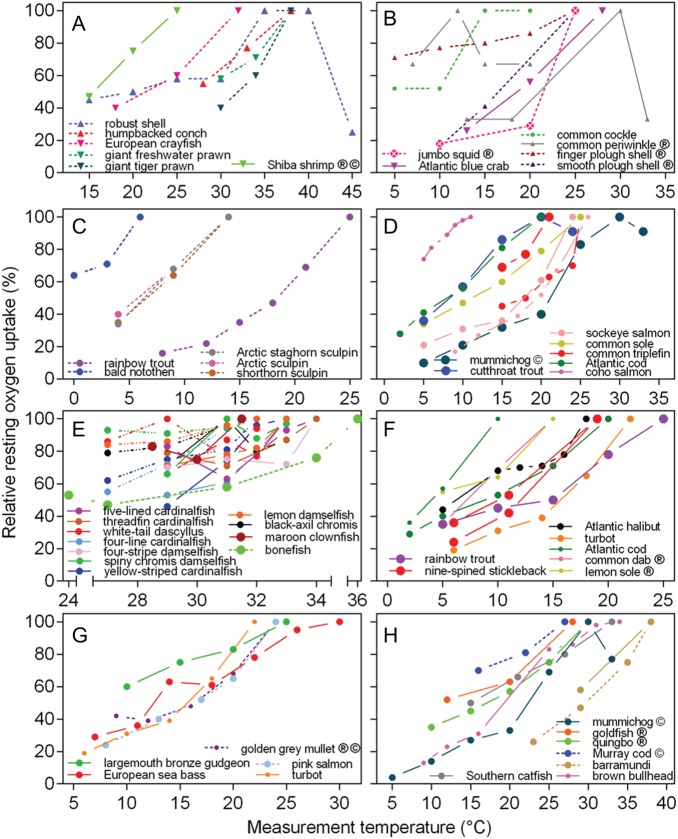
Relative resting oxygen uptake as a function of temperature in different invertebrates and teleost fish. Resting oxygen uptake was set to 100% at the temperature where it was highest. Data are from the same data sets and grouped as in the panels of Fig. 2. Different symbols represent gastropods (upright triangles), crustaceans (inverted triangles), bivalves (hexagons), cephalopods (circled crosses) and teleost fish (circles), because these were the only taxonomic groups for which data were available. Continuous lines represent acclimated individuals (minimum of 7 days at each temperature), whereas dashed lines represent acute measurements (from 30 min to 5.5 days). Multiple lines of the same colours represent different studies on the same species or studies on different populations of the same species. Symbol size reflects sample size at each temperature (<5 = small, 5–10 = medium and >10 = large). For most species, the temperature range over which aerobic scope was measured is representative for the temperatures at which the species occurs, or even up to critical temperatures. Species for which the relevance of the tested temperature range is unclear are indicated by ‘•’. Species for which routine rather than resting oxygen uptake was measured are indicated by ‘©’. The complete data set and reference details are available in Supplementary Table ST1.

**Figure 4:**
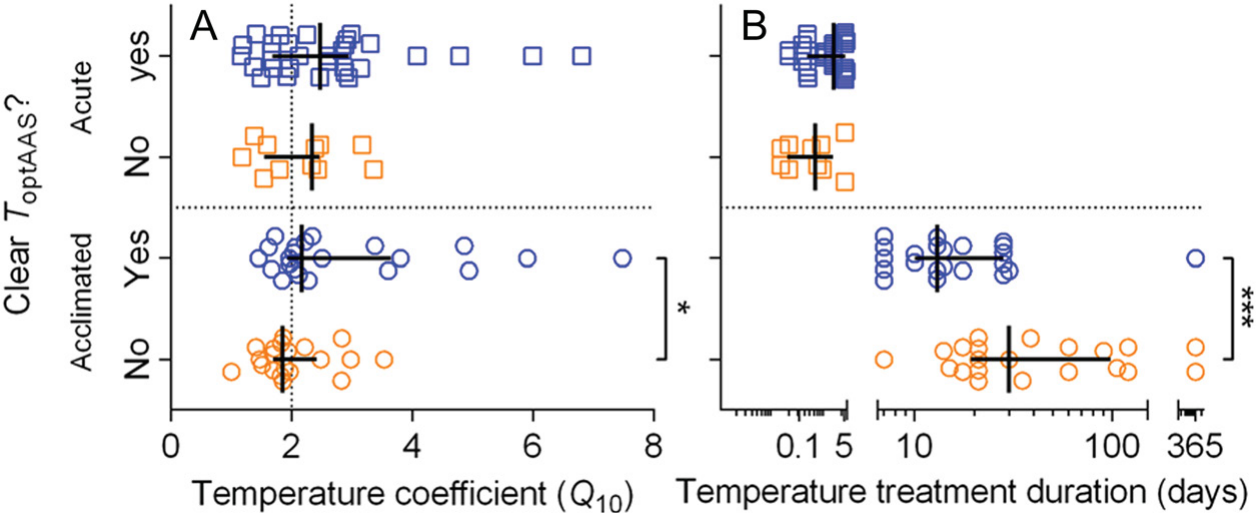
Effect of temperature coefficient (*Q*_10_) and acclimation time on the presence (yes) or absence (no) of an optimal temperature (*T*_optAAS_) for absolute aerobic scope. (**A**) Presence or absence of *T*_optAAS_ as a function of *Q*_10_ for resting oxygen uptake. (**B**) Presence or absence of *T*_optAAS_ as a function of the duration of temperature treatment in studies using acute treatment and acclimation. Continous lines indicate median ± interquartile range. Note the logarithmic scale in (B). Asterisk indicates significant difference in *Q*_10_ and acclimation duration, in studies with different outcome for acclimated species (*P < 0.05, ***P < 0.001; see ‘Results’ for details).

As expected, the relative resting oxygen uptake ($R\dot{M}O_{2\mathrm{rest}}$) increased over the entire temperature range in most species, both invertebrates (Fig. 3A and B) and fish (Fig. 3C–H). In robust shell (*Littoraria undulata*; Fig. 3A), common cockle (Fig. 3B) and mummichog (*Fundulus heteroclitus*; Fig. 3D and H), $R\dot{M}O_{2\mathrm{rest}}$ declined at the highest temperature. The *Q*_10_ was between 1 and 3 in most studies and was slightly, but significantly, lower in data sets on acclimated individuals in which a *T*_optAAS_ was absent (i.e. RAS never decreased below 90%; Fig. 4A; Mann–Whitney *U*-test, *P* = 0.0246). The highest *Q*_10_ values, above 4 and as high as 7, were found in the warmest coral reef fish populations (Papa New Guinea and Lizard Island), such as yellowstriped cardinalfish (*Ostorhinchus cyanosoma*), fourline cardinalfish (*Ostorhinchus doederleini*), lemon damsel (*Pomacentrus moluccensis*) and black-axil chromis (*Chromis atripectoralis*), regardless of acclimation time. The cardinalfishes were also the ones showing the largest decrease in RAS (Fig. 2E). Overall, there was no difference in the outcome of the studies (presence vs. absence of a thermal optimum for aerobic scope, *T*_optAAS_) between acute and acclimated (Fisher’s exact test, *P* = 0.345). However, the mean duration in studies on acclimated individuals where *T*_optAAS_ was absent was significantly longer (Fig. 4B; Mann–Whitney *U*-test, *P* = 0.0004) than in the studies in which a *T*_optAAS_ was identified. 

**Figure 5:**
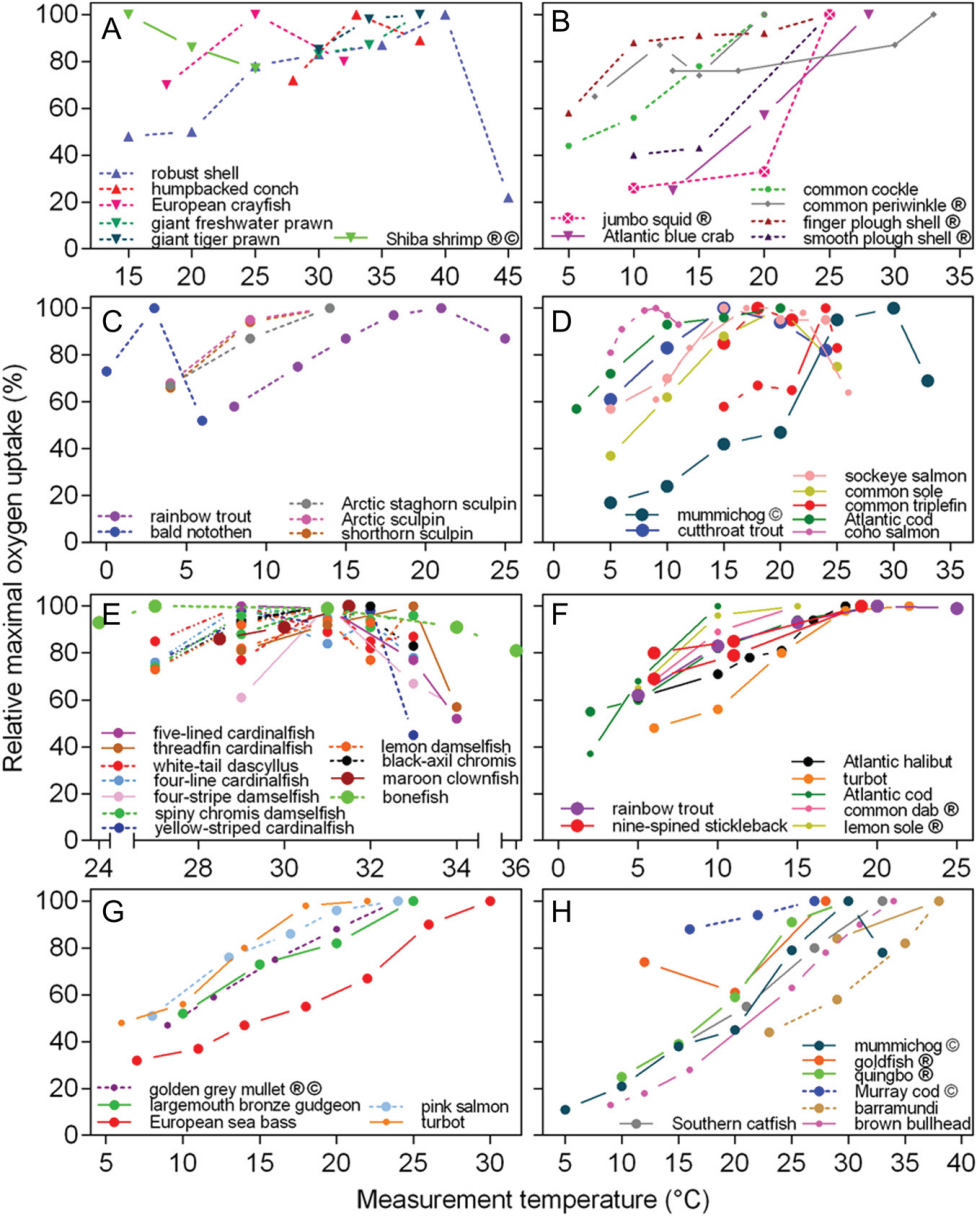
Relative maximal oxygen uptake as a function of temperature in different invertebrates and teleost fish. Maximal oxygen uptake was set to 100% at the temperature where it was highest. Data are from the same data sets and grouped as in the panels of Fig. 2. Different symbols represent gastropods (upright triangles), crustaceans (inverted triangles), bivalves (hexagons) and cephalopods (circled crosses) and teleost fish (circles). Continuous lines represent acclimated individuals (minimum of 7 days at each temperature), whereas dashed lines represent acute measurements (from 30 min up to 5.5 days). Multiple lines of the same colours represent different studies on the same species or studies on different populations of the same species. Symbol size reflects the sample size at each temperature (<5 = small, 5–10 = medium and >10 = large). For most species, the temperature range over which aerobic scope was measured is representative for the temperatures at which the species occurs, or even up to critical temperatures. Species for which the relevance of the tested temperature range is unclear are indicated by ‘•’. Species for which routine rather than resting oxygen uptake was measured are indicated by ‘©’. The complete data set and reference details are available in Supplementary Table ST1.

**Figure 6:**
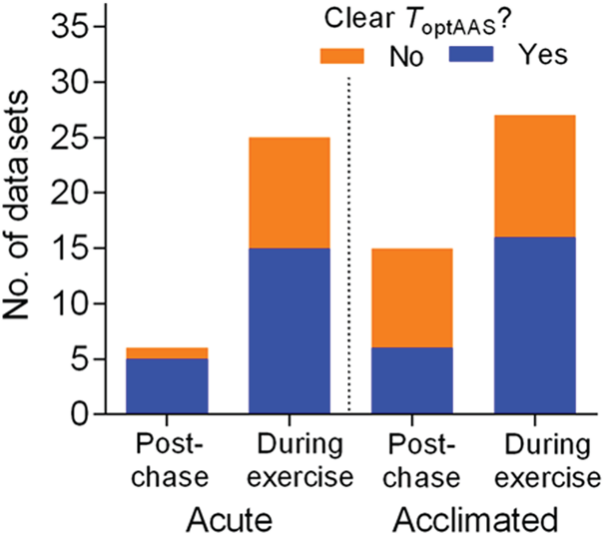
Effect of methodology on the presence or absence of an optimal temperature for absolute aerobic scope (*T*_optAAS_). Data are the number of data sets with absence or presence of a *T*_optAAS_ (‘no’ or ‘yes’) grouped by methodology (‘acute’ or ‘acclimated’, and ‘post-chase’ or ‘exercise’). There was no significant difference in the proportion of ‘yes’ and ‘no’ outcomes between the two methods in either acute or acclimated studies (see Results for details).

Unlike $R\dot{M}O_{2\mathrm{rest}}$, but similar to RAS, there were different responses of the relative maximal oxygen uptake ($R\dot{M}O_{2\max}$) with increased temperature. In most species of both invertebrates (Fig. 5A and B) and fish (Fig. 5C–H), $R\dot{M}O_{2\max}$ increased over most of the temperature range measured, showing no or only a minor decline at the upper temperature. Other species showed declines in $R\dot{M}O_{2\max}$ above mid-range temperatures (Fig. 5D). The coral reef fishes (Fig. 5E) again showed the most pronounced declines, despite having a much narrower thermal range, and this was also the group that showed the largest declines in RAS (Fig. 2E). When studies were divided into ones where $\dot{M}O_{2\max}$ was measured immediately after chasing or other exhaustive exercise (‘post-chase’) and ones where $\dot{M}O_{2\max}$ was measured during swimming or other maximal activity (‘during exercise’; Fig. 6), there was no difference in the outcome (presence vs. absence of *T*_optAAS_), in studies using either acute exposure (Fisher’s exact test, *P* = 0.383) or acclimation (Fisher’s exact test, *P* = 0.336). 

### Isolated and combined effects ’of temperature and CO_2_

**Figure 7:**
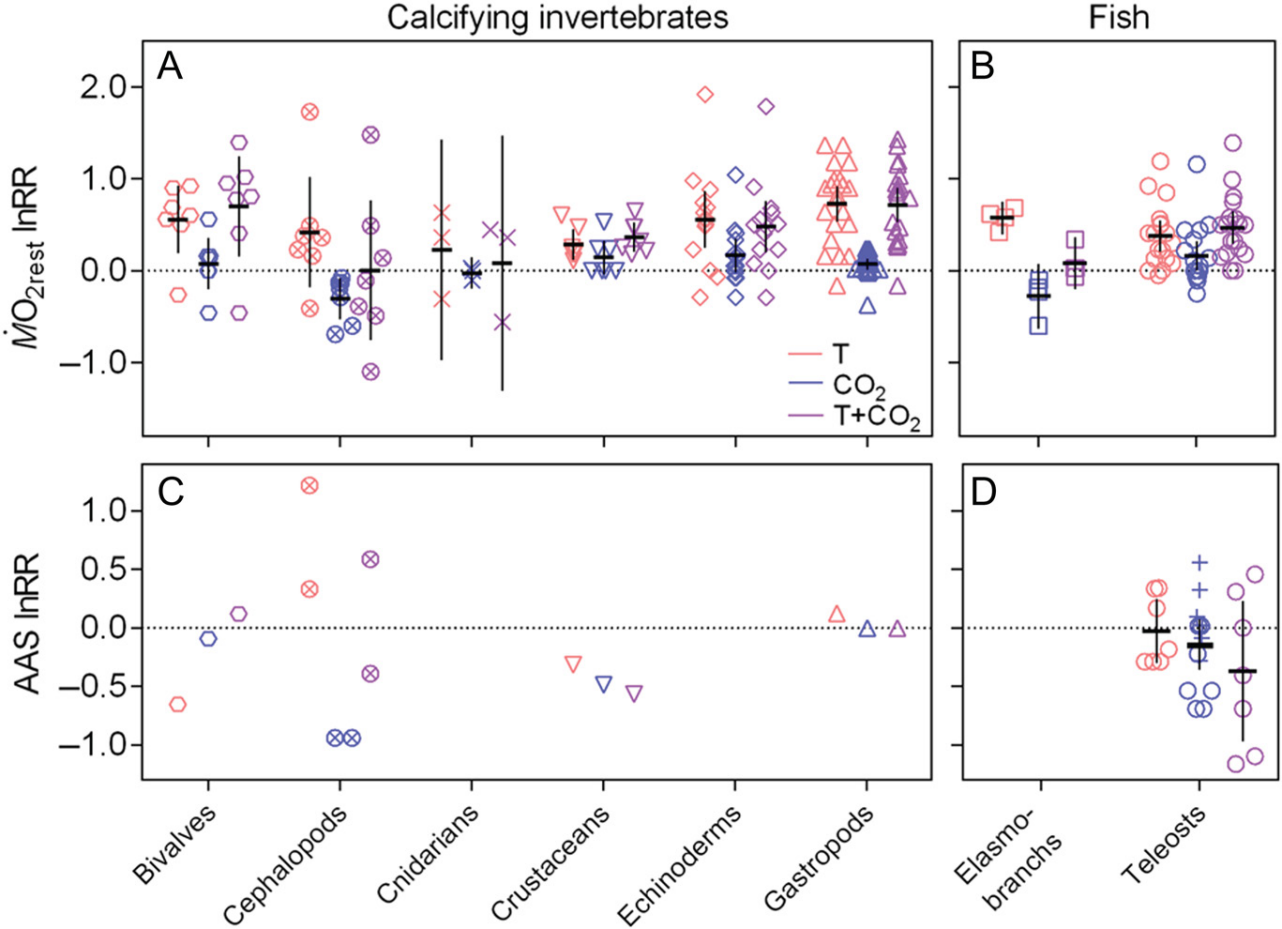
Isolated and combined effects of elevated temperature and partial pressure of CO_2_ (*P*CO_2_) on resting oxygen uptake (MO2rest) and absolute aerobic scope (AAS). Data are log response ratios (lnRR) of $MO_{2\mathrm{rest}}$ (**A** and **B**) and AAS (**C** and **D**) in marine invertebrates (A and C) and fish (B and D) to elevated temperature alone (red symbols), elevated *P*CO_2_ alone (blue symbols) and the combined treatment (purple symbols). Different shapes indicate different taxonomic groups (upright triangle, gastropod; circled cross, cephalopod; hexagon, bivalve; inverted triangle, crustacean; diamond, echinoderm; cross, cnidarian; square, elasmobranch; and circle, teleost). Note that the invertebrate species are all calcifying because there were no comparable data available for non-calcifying species. Continous lines indicate mean ± 95% confidence intervals. In (D), lnRR from studies investigating CO_2_ only (indicated by plus signs) have been added to illustrate the variation in responses, because this is not reflected in the studies having investigated both temperature and CO_2_. The complete data set and reference details are available in Supplementary Table ST3_MO2rest and ST3_AAS.

The mean log response ratio for the isolated effect of temperature (lnRR*_T_*) was significantly larger than zero (as judged by the 95% CI) in both calcifying invertebrates (Fig. 7A; except cephalopods and cnidarians, the groups with fewest studies) and fish (Fig. 7B), although the magnitude of the effect varied (mostly in invertebrates), and there were cases where $\dot{M}O_{2\mathrm{rest}}$ was unchanged. Only in invertebrates were reductions in $\dot{M}O_{2\mathrm{rest}}$ observed with elevated temperature. There was no clear distinction in responses between different taxonomic groups, except that response magnitude and variation seemed smaller within the crustaceans. Generally, the effects of CO_2_ on $\dot{M}O_{2\mathrm{rest}}$ were smaller than the effects of temperature, and more evenly distributed between positive and negative effects, generally resulting in a mean lnRR_CO2_ that was not different from zero. Exceptions to this included cephalopods and elasmobranchs, where $\dot{M}O_{2\mathrm{rest}}$ was mostly reduced (note that all were non-adults in these two groups), and crustaceans and echinoderms, where $\dot{M}O_{2\mathrm{rest}}$ increased in most cases. In both invertebrates (except cephalopods) and teleosts, the combined effect of temperature and CO_2_ on $\dot{M}O_{2\mathrm{rest}}$ resembled the isolated effect of temperature. 

The lnRR for the isolated effect of temperature on AAS in both calcifying invertebrates (Fig. 7C) and fish (Fig. 7D) was variable. In contrast, CO_2_ seemed to affect the AAS of invertebrates negatively in some cases, or not at all, whereas the response was more variable for fish (at least if the studies that investigated only CO_2_ are also included). For invertebrates, the combined effect of temperature and CO_2_ again resembled that of temperature, whereas it was more variable for fish.

### Examination of the variability ’in responses to CO_2_

**Figure 8:**
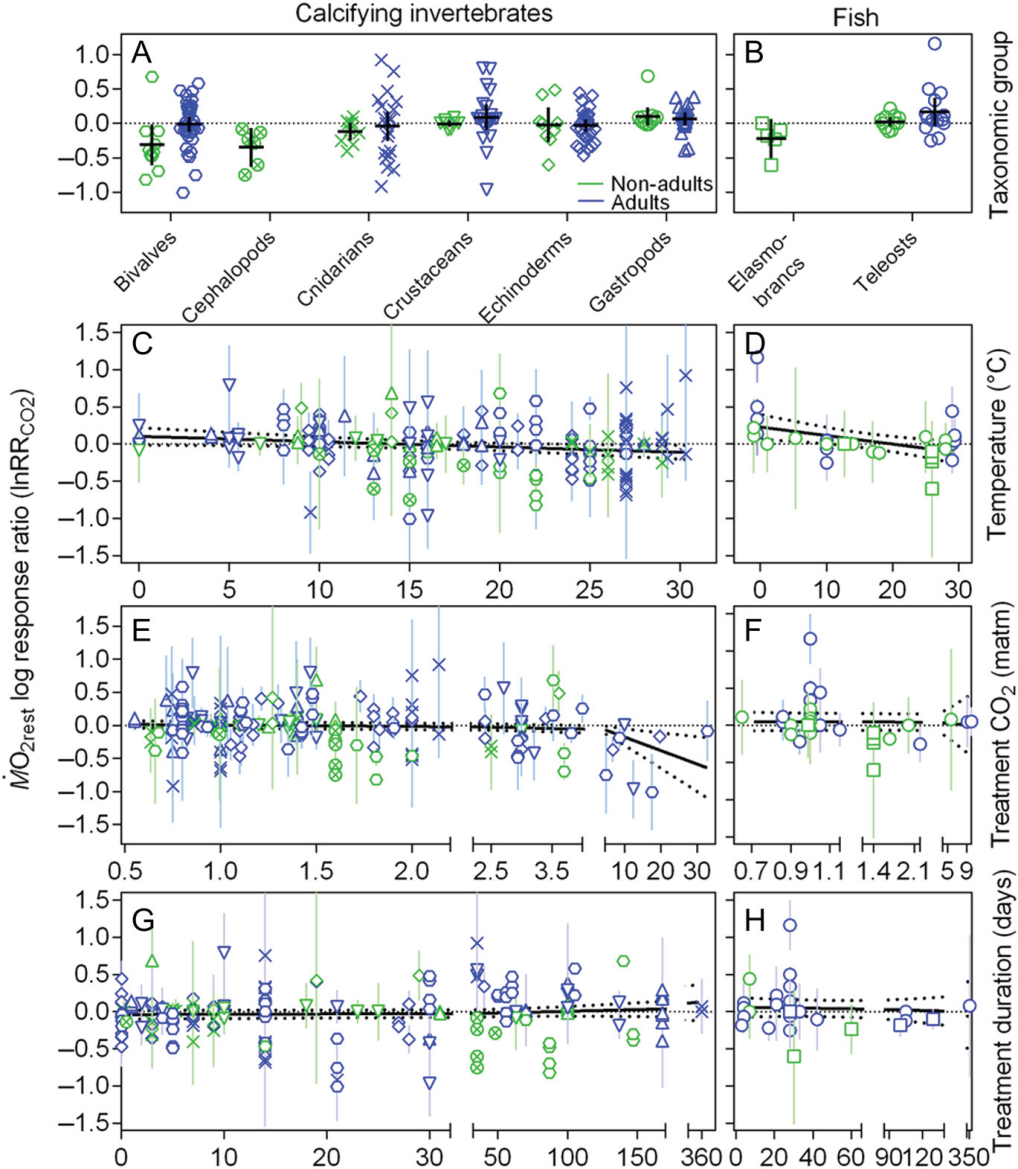
Effect of elevated CO_2_ on resting oxygen uptake ($\dot{M}O_{2\mathrm{rest}}$). Data are log response ratios to CO_2_ (lnRR_CO2_) of marine invertebrates (**A**, **C**, **E** and **G**) and teleosts and elasmobranchs (**B**, **D**, **F** and **H**) in non-adults (green) and adults (blue) of different taxonomic groups (upright triangle, gastropod; circled cross, cephalopod; hexagon, bivalve; inverted triangle, crustacean; diamond, echinoderm; cross, cnidarian; square, elasmobranchs; and circle, teleost). Data are also depicted as a function of the temperature at which the experiment was conducted (C and D), as a function of the *P*CO_2_ used as the experimental treatment (E and F) and as a function of the duration of the CO_2_ exposure (G and H). Note that the invertebrate species are all calcifying because there were no comparable data available for non-calcifying species. Continous lines indicate mean ± 95% confidence interval (A and B) or fitted lines from linear regression, with dotted lines representing the 95% confidence interval (C–H) (see Results for details). The complete data set and reference details are available in Supplementary Table ST2_MO2rest.

The effect of CO_2_ (lnRR_CO2_) on $\dot{M}O_{2\mathrm{rest}}$ tended to be more variable in adults than non-adults of both calcifying invertebrates (Fig. 8A) and fish (Fig. 8B), although data were lacking for adult cephalopods and elasmobranchs. Overall, differences in life stage explained only 1.75% of the total variation (two-way ANOVA, *F*_1,86_ = 3.58, *P* = 0.060). The lnRR_CO2_ was dominantly negative in non-adult compared with adult bivalves (Sidak’s multiple comparisons test, *P* = 0.050) but did not differ between life stages of the other taxonomic groups (*P* > 0.8 for all). The difference between the taxonomic groups explained only 6.92% of the total variation (two-way ANOVA, *F*_5,186_ = 2.84, *P* = 0.017), as lnRR_CO2_ of non-adult bivalves was more negative than that of non-adult gastropods (Sidak’s multiple comparisons test, *P* = 0.047) but did not differ between other groups of either non-adults (*P* = 0.223 for teleosts, *P* > 0.5 for all others) or adults (*P* > 0.5 for all). 

Overall, temperature did not explain much of the variation in lnRR_CO2_ of either invertebrates (Fig. 8C) or fish (Fig. 8D). There was a weak positive relationship between lnRR_CO2_ and the temperature at which the experiment was conducted in cnidarians (Pearson correlation, *r* = 0.456; linear regression, *R*^2^ = 0.208, *P* = 0.011) and a weak negative relationship in echinoderms (Pearson correlation, *r* = −0.361; linear regression, *R*^2^ = 0.131, *P* = 0.039). When data were pooled across groups and life stages, there was a weak negative relationship between lnRR_CO2_ and temperature in invertebrates (Pearson correlation, *r* = −0.170; linear regression, *R*^2^ = 0.025, *P* = 0.029) and a slightly stronger negative relationship in fish (Pearson correlation, *r* = −0.455; linear regression, *R*^2^ = 0.207, *P* = 0.010).

The treatment *P*CO_2_ did not explain much of the variation in lnRR_CO2_ of either invertebrates (Fig. 8E) or fish (Fig. 8F), because there was only a weak negative relationship in crustaceans (Pearson correlation, *r* = −0.494; linear regression, *R*^2^ = 0.244, *P* = 0.008) and in invertebrates altogether (Pearson correlation, *r* = −0.202; linear regression, *R*^2^ = 0.041, *P* = 0.008), but not in fish (Pearson correlation, *r* = −0.040; linear regression, *R*^2^ = 0.002, *P* = 0.830). Likewise, lnRR_CO2_ seemed to be unrelated to the duration of the CO_2_ exposure in both invertebrates (Fig. 8G; Pearson correlation, *r* = 0.089; linear regression, *R*^2^ = 0.008, *P* = 0.242) and fish (Fig. 8H; *r* = −0.086; *R*^2^ = 0.007, *P* = 0.644).

**Figure 9:**
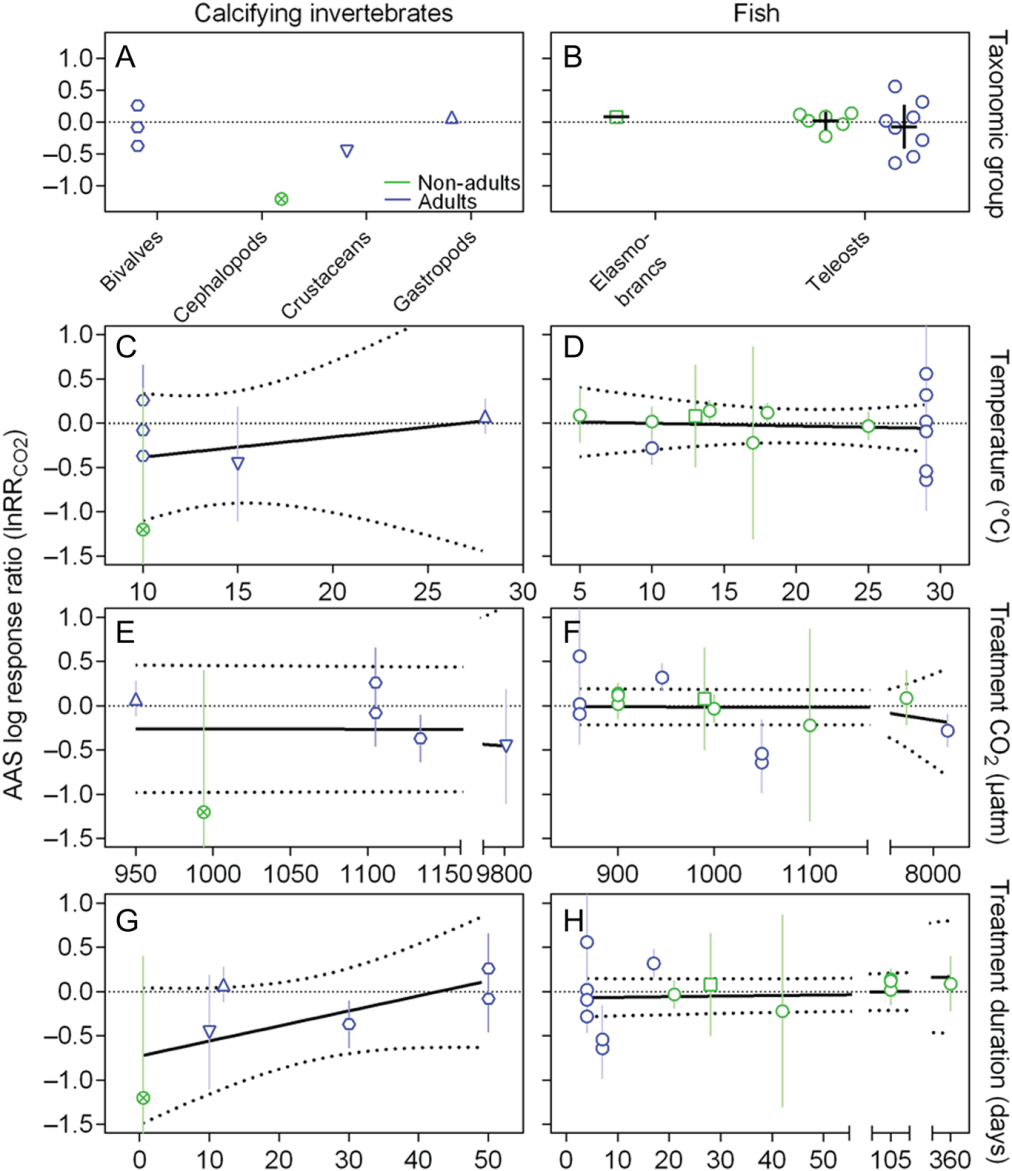
Effect of elevated CO_2_ on absolute aerobic scope (AAS). Data are log response ratios to CO_2_ (lnRR_CO2_) of calcifying invertebrates (**A**, **C**, **E** and **G**) and teleosts and elasmobranchs (**B**, **D**, **F** and **H**) in non-adults (green) and adults (blue) of different taxonomic groups (inverted triangle, crustacean; upright triangle, gastropod; hexagon, bivalve; circled cross, cephalopod; square, elasmobranch; and circle, teleost). Data are also depicted as a function of the temperature at which the experiment was conducted (C and D), as a function of the *P*CO_2_ used as the experimental treatment (E and F) and as a function of the duration of the CO_2_ exposure (G and H). Note that the invertebrate species are all calcifying because there were no comparable data available for non-calcifying species. Continous lines indicate mean ± 95% confidence interval (A and B) or fitted lines from linear regression, with dotted lines representing the 95% confidence interval (C–H) (see Results for details). The complete data set and reference details are available in Supplementary Table ST2_AAS.

In the studies on the effect of CO_2_ on AAS invertebrates, only one was conducted on a non-adult life stage (jumbo squid, *Dosidicus gigas*), and in this study elevated CO_2_ affected AAS negatively, whereas the effect on adults of other groups varied (Fig. 9A). In fish, the non-adult life stages appeared mostly unaffected by elevated CO_2_, whereas the responses in adults were both positive and negative (Fig. 9B), although data for adult elasmobranchs were not available. The difference between non-adult and adult teleosts was not significant (Student’s unpaired *t*-test, *P* = 0.610). There was no overall relationship between the effect of CO_2_ on AAS and the temperature at which the experiment was conducted in either invertebrates (Fig. 9C; Pearson correlation, *r* = 0.316; linear regression, *R*^2^ = 0.100, *P* = 0.542) or fish (Fig. 9D; *r* = −0.083; *R*^2^ = 0.007, *P* = 0.778). Likewise, was there no relationship with the treatment *P*CO_2_ in either invertebrates (Fig. 9E; Pearson correlation, *r* = −0.151; linear regression, *R*^2^ = 0.023, *P* = 0.776) or fish (Fig. 9F; *r* = −0.165; *R*^2^ = 0.027, *P* = 0.572). In invertebrates, there was a small tendency for the effect to diminish with increased duration of the exposure (Fig. 9G; Pearson correlation, *r* = 0.699; linear regression, *R*^2^ = 0.489, *P* = 0.122), but this was not the case in fish (Fig. 9H; *r* = 0.200; *R*^2^ = 0.040, *P* = 0.493). 

**Figure 10:**
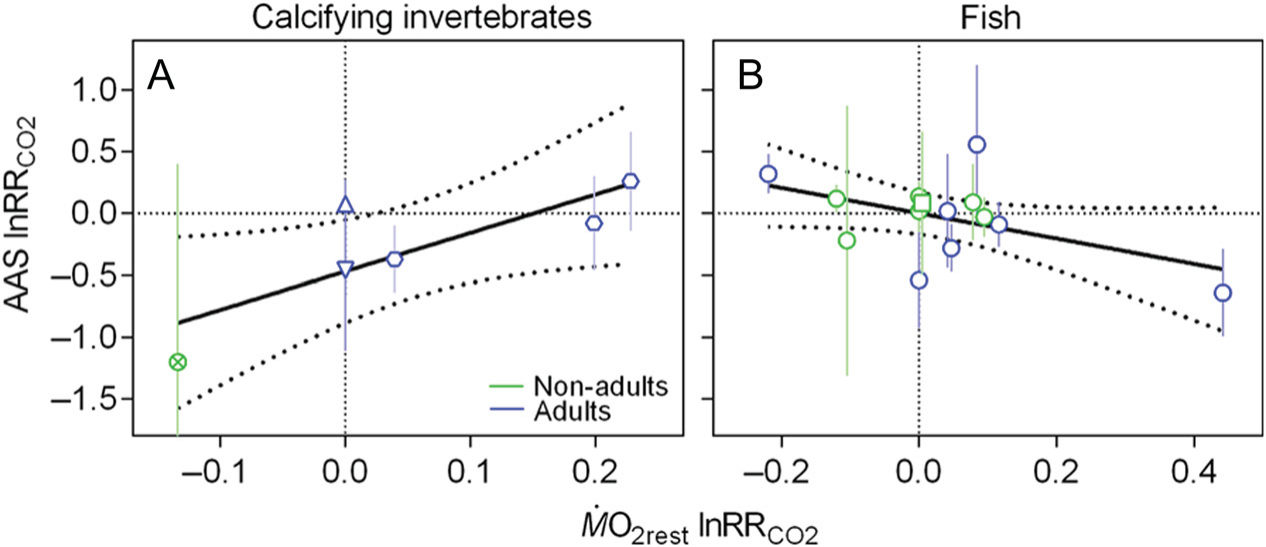
The effect of elevated *P*CO_2_ on absolute aerobic scope (AAS) as a function of the effect on resting oxygen uptake ($MO_{2\mathrm{rest}}$). Data are log response ratios to CO_2_ (lnRR_CO2_) with 95% confidence intervals in calcifying invertebrates (**A**) and teleosts and elasmobranchs (**B**) of both non-adult (green) and adult (blue) life stages. Different symbols indicate different taxonomic groups (upright triangle, gastropod; inverted triangle, crustacean; circle, teleosts; and square, elasmobranch). Note that the invertebrate species are all calcifying because there were no comparable data available for non-calcifying species. The continous lines are fitted by linear regression, and dotted lines represent the 95% confidence interval (see Results for details).

In invertebrates (Fig. 10A), the relationship between the effect of CO_2_ on $\dot{M}O_{2\mathrm{rest}}$ (lnRR_MO2rest_) and the effect of CO_2_ on AAS (lnRR_AAS_) was positive and marginally significant (linear regression, *R*^2^ = 0.665, *P* = 0.0479). The relationship appeared to be opposite for fish (Fig. 10B; linear regression, *R*^2^ = 0.242, *P* = 0.0741), although this pattern was driven by one case (yellowstriped cardinalfish) where $\dot{M}O_{2\mathrm{rest}}$ was strongly elevated by CO_2_ exposure, and the relationship was not significant when this point was excluded (linear regression, *R*^2^ = 0.011, *P* = 0.736). 

**Figure 11:**
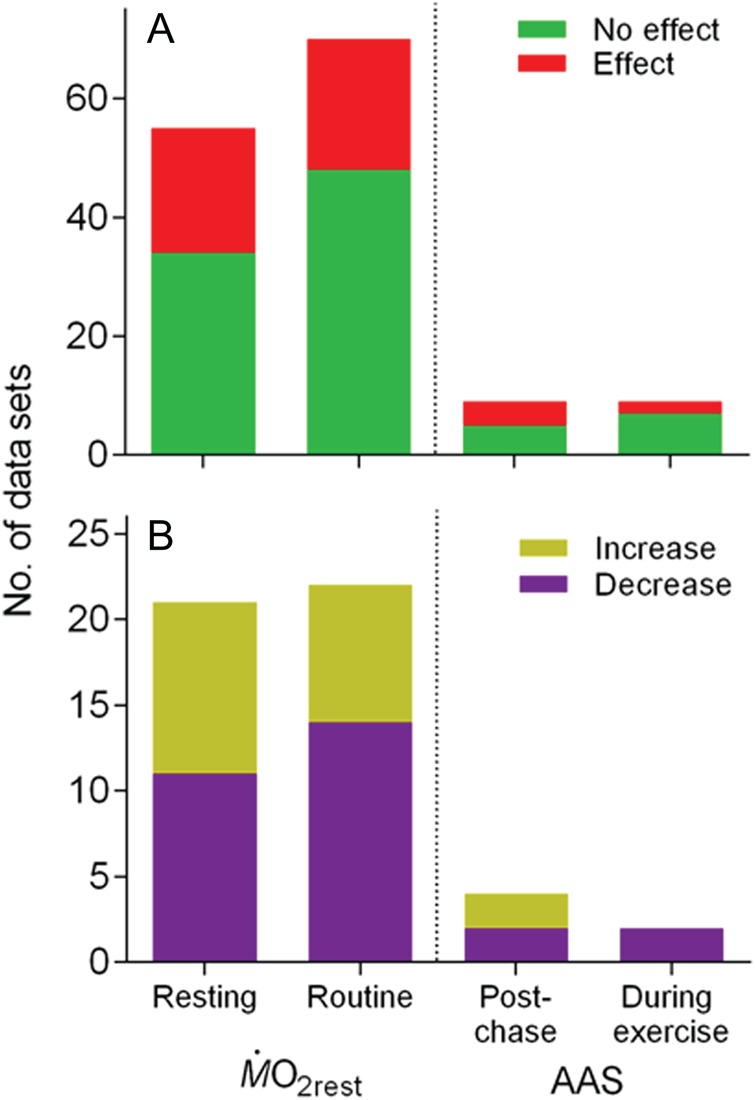
Effect of methodology on the outcome of CO_2_ treatment on resting oxygen uptake ($\dot{M}O_{2\mathrm{rest}}$) and absolute aerobic scope (AAS). Data are number of data sets grouped according to whether resting or routine oxygen uptake was measured (for $\dot{M}O_{2\mathrm{rest}}$), and whether maximal oxygen uptake (for AAS) was measured after chasing (post-chase) or during exercise. All taxonomic groups are included. (**A**) The number of data sets showing an effect (95% confidence interval of lnRR not overlapping zero) vs. no effect (95% confidence interval of lnRR overlapping zero). (**B**) The number of data sets (of the ones with an effect) showing a decrease vs. an increase. There were no significant differences in the proportions (see Results for details).

When dividing studies according to the methodology used to estimate $\dot{M}O_{2\mathrm{rest}}$ (‘resting’ vs. ‘routine’) and AAS (‘post-chase’ vs. ‘during exercise’) and comparing the outcome (effect vs. no effect) of elevated-CO_2_ treatment (Fig. 11A), there was no difference in the proportions for either $MO_{2\max}$ (Fisher’s exact test, *P* = 0.454) or AAS (*P* = 0.620). Likewise, in studies were an effect was found was there no differences in the proportion finding a decrease vs. an increase (Fig. 11B) when comparing either ‘resting’ with ‘routine’ (Fisher’s exact test, *P* = 0.543) or ‘post-chase’ with ‘during exercise’ (*P* = 0.467). 

### Nature of the interaction between ’temperature and CO_2_

**Figure 12:**
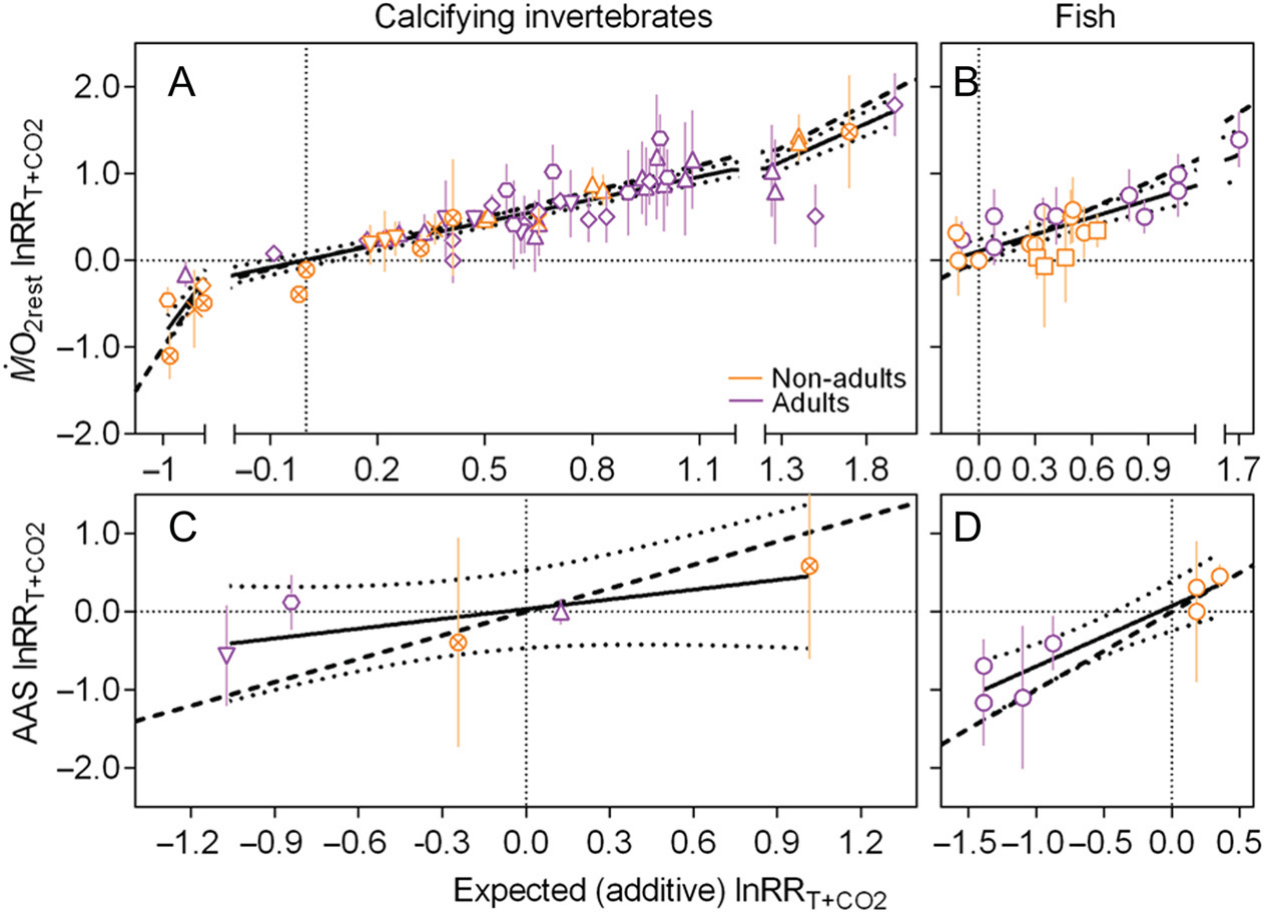
Measured combined log response ratio as a function of the expected additive log response ratio. Data are log response ratios (lnRR*_T_*_+CO2_ with 95% confidence intervals) for resting oxygen uptake ($\dot{M}O_{2\mathrm{rest}}$; **A** and **B**) and absolute aerobic scope (AAS; **C** and **D**) of both non-adult (orange) and adult (purple) life stages in calcifying invertebrates (A and C) and fish (B and D). Different symbols indicate different taxonomic groups (upright triangle, gastropod; inverted triangle, crustacean; circled cross, cephalopod; hexagon, bivalve; diamond, echinoderm; cross, cnidarians; circle, teleost; and square, elasmobranch). Note that the invertebrate species are all calcifying because there were no comparable data available for non-calcifying species. The continous lines are fitted by linear regression, and the dotted lines represent the 95% confidence interval. The dashed 1/1 line is where the measured lnRR*_T_*_+CO2_ equals the expected additive lnRR.

For $\dot{M}O_{2\mathrm{rest}}$, there was a positive significant relationship between measured and expected additive lnRR for both invertebrates (Fig. 12A; linear regression, *R*^2^ = 0.838, *P* < 0.0001, measured-lnRR*_T_*_+CO2_ = 0.87 × expected-lnRR*_T_*_+CO2_ + 0.007) and fish (Fig. 12B; *R*^2^ = 0.657, *P* < 0.0001, measured-lnRR*_T_*_+CO2_ = 0.66 × expected-lnRR*_T_*_+CO2_ + 0.104). In both invertebrates and fish, the slope was significantly smaller than 1 (non-linear regression fit of straight line, rejecting the null hypothesis that slope = 1.0 with *P* = 0.0142 and *P* = 0.004, respectively), indicating—if anything—antagonistic rather than synergistic interactions between temperature and CO_2_. This was also the case when different taxonomical groups were analysed individually, so there did not appear to be a particular correlation of taxonomic group or life stage with the nature of the interaction. 

For AAS, there was no significant relationship between measured and expected additive lnRR for invertebrates (Fig. 12C; *R*^2^ = 0.581, *P* = 0.134, measured-lnRR*_T_*_+CO2_ = 0.415 × expected-lnRR*_T_*_+CO2_ + 0.034), but there was a positive significant relationship for vertebrates (Fig. 12D; *R*^2^ = 0.870, *P* = 0.002, measured-lnRR*_T_*_+CO2_ = 0.776 × expected-lnRR*_T_*_+CO2_ + 0.076). In both invertebrates and vertebrates, the slope was smaller but not significantly different from 1 (non-linear regression fit of straight line, not rejecting the null hypothesis that slope = 1.0 with *P* = 0.155 and *P* = 0.064, respectively), indicating—if anything—an additive rather than synergistic interaction. As for $\dot{M}O_{2\mathrm{rest}}$, there did not appear to be a particular correlation between taxonomic group and the nature of the interaction, although the smaller sample size for AAS of invertebrates and the lack of data on elasmobranchs should be kept in mind. In contrast, it is noteworthy that for the teleosts investigated, the juvenile life stages seemed to display additive effects, whereas the adults showed more antagonistic effects, although this could also be a result of all the four adults being coral reef fish, whereas the juveniles were temperate species.

## Discussion

### Aerobic-scope curves across species and time frames

The diversity in the shape of the aerobic-scope curves, and particularly, the apparent absence of *T*_optAAS_ in many species, is difficult to reconcile with a single unifying model for how temperature affects respiratory variables in marine ectothermic animals. The main impression is that studies on the effect of temperature on AAS yield divergent results, and that this may be related to the diversity in physiology and habitats of the species, and to the time frame of the study. The present analysis therefore supports the responses outlined in the reviews by Clark *et al.* (2013) and Schulte (2015); that is, some species show an increase–optimum–decrease-type response, as predicted by the Fry paradigm and the OCLTT hypothesis, whereas others seem able to maintain or continue to increase AAS with rising temperature, and a clear *T*_optAAS_ is not evident. These patterns were observed in the data on both teleosts and invertebrates (of which only gastropods, crustaceans, cephalopods and bivalves have been investigated). For the species that show bell-shaped curves, the data support the widely accepted notion that thermal reaction norms reflect the variability of habitat temperatures (e.g. Tewksbury *et al.*, 2008), so that animals from variable temperate climates have broad thermal windows, whereas polar or equatorial species, e.g. the coral reef fish, display narrow windows.

As outlined in the Introduction, the underlying cause of the increase in AAS with temperature at the lower end of the thermal window must be that $\dot{M}O_{2\max}$ increases faster than $\dot{M}O_{2\mathrm{rest}}$ (Fig. 1A). Presumably, this difference arises because $\dot{M}O_{2\mathrm{rest}}$ increases owing to the temperature dependence of underlying chemical processes (following the Arrhenius equation), whereas $\dot{M}O_{2\max}$ can be enhanced actively by adjustments in the circulatory and respiratory systems, which benefits from increased temperature (e.g. Gräns *et al.*, 2014). At the other side of *T*_optAAS_, $\dot{M}O_{2\mathrm{rest}}$ is assumed to increase inexorably, virtually until the animal dies, while the cardiorespiratory system is unable to support further increases in $\dot{M}O_{2\max}$, resulting in a decrease in scope. The predicted increase in $\dot{M}O_{2\mathrm{rest}}$ is generally found in all species, as is the predicted initial increase in $\dot{M}O_{2\max}$, although the suggested eventual decline is not found in all species. Species with a temperature effect on $\dot{M}O_{2\mathrm{rest}}$ (high *Q*_10_), combined with an inability to increase $\dot{M}O_{2\max}$, may therefore be more likely to suffer from reductions in aerobic scope, whereas species with a more moderate *Q*_10_, and where the $\dot{M}O_{2\max}$ does not seem to be limited at high temperatures, will also show a continuous increase in AAS. Although the aerobic scope curve is obviously shaped by a combination of the temperature effect on $\dot{M}O_{2\mathrm{rest}}$ and $\dot{M}O_{2\max}$, the ultimate outcome seems to be most closely reflected by $\dot{M}O_{2\max}$. Importantly, the method used to estimate $\dot{M}O_{2\max}$ does not seem to influence the shape of the curve. Another methodological aspect that is important to keep in mind is the possibility that some species may increase spontaneous activity as temperature rises (Reynolds and Casterlin, 1982; Forstner and Wieser, 1990; Castonguay and Cyr, 1998; Stoner *et al*., 2006), whereas others may not (Peterson and Anderson, 1969; Stevens and Fry, 1972; Schurmann and Steffensen, 1994; Crocker and Cech, 1997; Johansen *et al*., 2014). If routine rather than resting $\dot{M}O_{2}$ levels are measured and used as $\dot{M}O_{2\mathrm{rest}}$ to calculate AAS, one may observe an increase in $\dot{M}O_{2}$ caused by activity rather than an increase in basal oxygen demand, and hence a decrease in aerobic scope that reflects differences in activity rather than aerobic capacity. The majority of studies included in the present analysis measured $\dot{M}O_{2\mathrm{rest}}$ in conditions that can be considered resting, but there was no obvious bias in the outcome of the few studies using routine rates, because two studies found AAS to increase continually with temperature (Clark *et al*., 2005; Vagner *et al*., 2015), whereas three studies found a clear *T*_optAAS_ (Lee *et al*., 2003; Dissanayake and Ishimatsu, 2011; Healy and Schulte, 2012a).

There is no doubt that an increase in $\dot{M}O_{2\mathrm{rest}}$ and limitation of $\dot{M}O_{2\max}$ are likely within an acute scenario (minutes, hours and maybe days, at a new temperature), where the animal is not given time to adjust its metabolic processes and thereby their costs. This is also supported by the data, as several of the acutely performed measurements of AAS reveal a typical bell-shaped increase–optimum–decrease-type response [robust shell (*Littoraria undulata*; Patnaik *et al.*, 1985); bald notothen (Lowe and Davison, 2006); sockeye salmon (*Oncorhynchus nerka*; Eliason *et al.*, 2011); shorthorn sculpin (*Myoxocephalus scorpius*), Arctic sculpin (*Myoxocephalus scorpioides*) and Arctic staghorn sculpin (*Gymnocanthus tricuspis*; Seth *et al*., 2013); goldfish (*Carassius auratus*; Ferreira *et al*., 2014); rainbow trout (*Oncorhynchus mykiss*; Chen *et al*., 2015); European crayfish (*Astacus astacus*) and giant tiger prawn (*Penaeus monodon*; Ern *et al.*, 2015); humpback conch (*Gibberulus gibberulus gibbosus*; Lefevre *et al.*, 2015); and bonefish (*Albula vulpes*; Nowell *et al*., 2015)]. Intriguingly, a study on a high-Arctic population of blue mussel (*Mytilus edulis*) acclimated to 1°C (Thyrring *et al.*, 2015) found that AAS during an acute temperature challenge was highest at 7°C, even though this population probably never experiences a temperature higher than 5°C during the warmest month of the year. Likewise, there are species that under acute temperature challenges continue to increase AAS up to *CT*_max_ [barramundi (*Lates calcarifer*; Norin *et al.*, 2014); and pink salmon (*Oncorhynchus gorbuscha*; Clark *et al.*, 2011)] or up to the highest temperatures they may experience in their habitats [jumbo squid (Rosa and Seibel, 2008); Murray cod (*Maccullochella peelii*; Clark *et al.*, 2005); and golden grey mullet (*Liza aurata*; Vagner *et al.*, 2015)]. These cases are difficult to reconcile with the view that a limitation of the capacity for oxygen delivery is the main driver of thermal tolerance.

The concept of OCLTT has, however, not only been applied to acute circumstances, but is also suggested to explain species distribution limits and hence predict changes in distributions under global warming and ocean acidification (e.g. Bozinovic and Pörtner, 2015), processes occurring at a much slower pace than what can be mimicked in the laboratory. This automatically introduces a contradiction with the well-established paradigm that animals, given enough time, will down-regulate their basal oxygen demand and may even compensate fully for the elevated temperature (Bullock, 1955; Segal, 1961; Hazel and Prosser, 1974; Johnston and Dunn, 1987; Sandblom *et al*., 2014; Seebacher *et al.*, 2015), presumably to minimize energy demand. Although this makes sense, at least if it is assumed that animals are generally energy restricted, it does conflict with the assumption that $\dot{M}O_{2\mathrm{rest}}$ increases exponentially, which contributes to the shape of the aerobic performance curve under the Fry paradigm and hence the OCLTT hypothesis. The ability to acclimate could thus explain why several species appear to show increases [common cockle (Newell, 1966); cutthroat trout (*Oncorhynchus clarkii*; Dwyer and Kramer, 1975); smooth plough shell (*Bullia rhodostoma*; Brown and da Silva, 1984); Atlantic blue crab (Booth and McMahon, 1992); European sea bass (*Dicentrarchus labrax*; Claireaux *et al.*, 2006); southern blue catfish (*Ictalurus meridionalis*; Pang *et al.*, 2010); largemouth bronze gudgeon (*Coreius guichenoti*; Tu *et al*., 2012); and nine-spined stickleback (*Pungitius sinensis*; Bruneaux *et al*., 2014)] or only minor decreases [sockeye salmon (Brett, 1964); common periwinkle (Newell and Pye, 1970, 1971); rainbow trout (Dickson and Kramer, 1971); finger plough shell (*Bullia digitalis*; Brown and da Silva, 1983); turbot (*Scophthalmus maximus*; Mallekh and Lagardere, 2002); mummichog (Healy and Schulte, 2012a); common triplefin (*Forsterygion lapillum*; Khan and Herbert, 2012; Khan *et al.*, 2014a); Atlantic halibut (*Hippoglossus hippoglossus*; Gräns *et al.*, 2014); salema and marbled spinefoot (Marras *et al*., 2015)] in AAS after acclimation to an increased temperature.

It is clear that acute studies can yield temperature response curves that are different from those obtained in the same species after acclimation to the different temperatures. This is, for example, illustrated by different studies on Atlantic cod (*Gadus morhua*). Sylvestre *et al*. (2007) found that RAS was already reduced to 78% at 13°C, whereas Tirsgaard *et al*. (2015a) found that RAS had not yet decreased at 15°C, in individuals of a similar size, after 2–3 weeks of acclimation. Perhaps even more extreme is the case of rainbow trout, where acute studies by Chen *et al*. (2015) showed severe decreases in RAS to 45% at 25°C, whereas Dickson and Kramer (1971) found RAS to be maintained at 94% after 2 weeks of acclimation at 25°C. The maximal AAS was similar in the two studies (550 and 498 mg O_2_ kg^−1^ h^−1^, respectively). In both Atlantic cod and rainbow trout, the *T*_optAAS_ with acute temperature changes coincided with the temperature at which the fish were acclimated. Another illustrative example of the importance of acclimation, and even transgenerational acclimation for $\dot{M}O_{2\mathrm{rest}}$ (and presumably also AAS), comes from rearing studies. In their first study, Donelson *et al*. (2011) showed that spiny chromis damselfish (*Acanthochromis polyacanthus*), reared from the larval stage (30 days post-hatch) at a 3°C higher temperature than the natural temperature in their habitat, partly compensated for the rise in $\dot{M}O_{2\mathrm{rest}}$ that was displayed by conspecifics acutely exposed to the same temperature. Subsequently, it was shown (Donelson *et al*., 2012) that offspring of spiny chromis damselfish kept at the +3°C regime also displayed $\dot{M}O_{2\mathrm{rest}}$ values that were fully compensated, revealing transgenerational acclimation. Overall, these examples and the present analysis show that studies of shorter duration (i.e. no or little acclimation) may be more likely to identify a *T*_optAAS_, indicating a difference in the physiological effects between acute and long-term exposures. Such a difference is obviously important to keep in mind when interpreting aerobic scope data and using it for modelling.

One can, of course, argue that studies failed to reveal a decline in AAS because temperatures were not high enough, i.e. if one had continued to increase the temperature one would eventually see a decline in AAS. But the majority of the studies finding that AAS does not decline have used a range of temperatures matching the distributional temperature range [except lemon sole (*Microstomus kitt*), common dab (*Limanda limanda*; Duthie, 1982); Atlantic cod (Claireaux *et al*., 2000); qingbo (*Spinibarbus sinensis*; Pang *et al.*, 2013); and goldfish (Ferreira *et al*., 2014)] and still show that AAS within these more ecologically relevant temperatures is not compromised. Thus, in these cases it is unlikely that AAS is the factor restricting the distribution of that species, and alternative mechanisms must be investigated to identify factors that can be used in the prediction of distributional changes with climate change. That is, even if AAS is not limited at the upper temperature range of a species, there may be other important performance measures that have a *T*_opt_, such as reproduction and growth. One example is the barramundi, where AAS is the same at 38°C as at 29°C (after acclimation), but where the optimal temperature for growth and even the preferred temperature is 31°C (Norin *et al*., 2014). In Atlantic cod, evolutionary bioenergetics modelling suggested AAS to be a poor predictor of optimal temperature for fitness as a whole (Holt and Jørgensen, 2015). Furthermore, a recent meta-analysis revealed that the acclimatory plasticity characteristic of aerobic metabolism is not observed to the same extent for lethal temperature limits (Gunderson and Stillman, 2015). For species living in environments with increased likelihood of short but extreme temperature peaks (e.g. tidepools and streams), a limited plasticity of critical temperature may be more important than AAS in determining future success, because extreme climatic events are expected to be more common in the future (Rahmstorf and Coumou, 2011; Fey *et al.*, 2015).

The number of examples that do not follow the predictions by the Fry paradigm, and where the OCLTT hypothesis is therefore not supported, has grown to a proportion that can hardly be classified as exceptions to a rule. But nonetheless, these cases cannot be considered as arguments for completely abandoning the concept either (or ‘throw out the baby with the bathwater’, as it was put by Farrell, 2016). It cannot be ruled out that OCLTT guides the performance of such animals that, even when given time to acclimate, still have a *T*_optAAS_, or at least a decline in AAS at elevated temperatures, also within the ecologically relevant temperature range [sockeye salmon (Lee *et al*., 2003); common sole (*Solea solea*; Lefrancois and Claireaux, 2003); bald notothen (Lowe and Davison, 2006); hapuku wreckfish (Khan *et al.*, 2014b); and various coral reef fish (Nilsson *et al*., 2009; Gardiner *et al.*, 2010; Johansen and Jones, 2011; Rummer *et al.*, 2014)]. In these cases, the acclimated response resembles the acute response, and this may be a direct result of an elevation in $\dot{M}O_{2\mathrm{rest}}$ and/or limitation of $\dot{M}O_{2\max}$ that cannot be alleviated through acclimation. This may even be the expected outcome for species that are adapted to a narrow temperature range and are lacking or have lost the genes necessary for acclimation outside this range. Many coral reef fish and polar species appear to be good examples of animals with a thermal history that has led to a narrow thermal window for physiological performance. A recent study found a good correlation between performance data from the field (activity and growth) and from the laboratory (AAS; Payne *et al.*, 2016), but notably, this correlation was obtained after excluding all the species where AAS did not decline at high temperature. In any case, finding that *T*_optAAS_ and *T*_optFIT_ correlate does not prove causal relationship between the two, and as such, a more mechanistic experimental approach is necessary to demonstrate OCLTT in a species (e.g. Healy and Schulte, 2012b; Overgaard *et al*., 2012; Ellis *et al*., 2013; Verberk *et al*., 2013; Wang *et al*., 2014; Brijs *et al*., 2015; Ern *et al*., 2015), even if evidence both for and against the predictions from the hypothesis exists.

### Does elevated CO_2_ in general cause resting oxygen uptake to increase?

Hypercapnia has for long been used in fish physiology research as a tool for studying the regulation of respiration and control of acid–base balance, and much of our basic understanding of pH regulation in fish is based on such experiments. These studies involved exposure to levels of CO_2_ many times higher than those relevant from an ocean acidification perspective. In some cases, these high CO_2_ levels interfered directly with ventilation (e.g. Kinkead and Perry, 1991; Crocker and Cech, 2002; Vulesevic *et al.*, 2006; Perry and Abdallah, 2012), and thereby, possibly $\dot{M}O_{2}$ and AAS. To evaluate the effects of future ocean acidification, it is much lower *P*CO_2_ levels that matter, because the current predictions are in the 1000 µatm range (Meinshausen *et al.*, 2011; Doney *et al.*, 2012).

It can be argued that CO_2_ exposure could cause an increase in $\dot{M}O_{2\mathrm{rest}}$, either through direct increases in costs associated with adjusting to elevated internal CO_2_, or alternatively, by inducing a general stress reaction. Indeed, for some animals in some conditions, $\dot{M}O_{2\mathrm{rest}}$ has been shown to be elevated significantly during prolonged CO_2_ exposure [yellowstriped cardinalfish (Munday *et al.*, 2009); Atlantic oyster (*Crassostrea virginica*; Beniash *et al*., 2010); Pacific oyster (*Crassostrea gigas*; Lannig *et al*., 2010); serpent star (*Ophiura ophiura*; Wood *et al.*, 2010); Schayer’s brittlestar (*Ophionereis schayeri*; Christensen *et al*., 2011); purple sea urchin (*Paracentrotus lividus*; Catarino *et al.*, 2012); dwarf cushion star (*Parvulastra exigua*; McElroy *et al.*, 2012); green sea urchin (*Strongylocentrotus droebachiensis*; Dorey *et al*., 2013); bald notothen, emerald rockcod (*Trematomus bernacchii*) and striped rockcod (*Trematomus hansoni*; Enzor *et al.*, 2013)]. Nevertheless, in most of the published experiments, elevated CO_2_ did not significantly affect $\dot{M}O_{2\mathrm{rest}}$ [e.g. Atlantic cod (Melzner *et al*., 2009; Tirsgaard *et al*., 2015b); an Arctic pteropod (*Limacina helicina*; Comeau *et al*., 2010); Shiba shrimp (*Metapenaeus joyneri*; Dissanayake and Ishimatsu, 2011); *Acesta excavata* (Hammer *et al*., 2011); Zhikong scallop (*Chlamys farreri*; Mingliang *et al*., 2011); burrowing shrimp (*Upogebia deltaura*; Donohue *et al*., 2012); various copepods (Li and Gao, 2012; Hildebrandt *et al*., 2014; Zervoudaki *et al*., 2014; Thor and Dupont, 2015); the bivalves *Chlamys nobilis*, *Perna viridis* and *Pinctada fucata* (Liu and He, 2012); marbled rockcod (*Notothenia rossii*; Strobel *et al*., 2012); Northern shrimp (*Pandalus borealis*; Arnberg *et al*., 2013); porcelain crab (*Petrolisthes cinctipes*; Carter *et al*., 2013); common starfish (*Asterias rubens*; Collard *et al*., 2013); hard-shelled clam (*Mercenaria mercenaria*; Dickinson *et al*., 2013; Matoo *et al*., 2013); the brittlestars *Ophiothrix fragilis* and *Amphiura filiformis* (Carey *et al*., 2014); the sea cucumbers *Holothuria parva* and *Holothuria scabra* (Collard *et al*., 2014); small-spotted catshark (*Scyliorhinus canicula*; Green and Jutfelt, 2014); Atlantic halibut (Gräns *et al*., 2014); European sea bass (Pope *et al*., 2014); white-spotted bamboo shark (*Chiloscyllium punctatum*; Rosa *et al*., 2014a); Pacific sea urchins (*Echinometra* sp. *A*; Uthicke *et al*., 2014); rainbow abalone (*Haliotis iris*; Cunningham *et al*., 2015); red drum (*Sciaenops ocellatus*; Esbaugh *et al*., 2016); various coral reef fish (Couturier *et al*., 2013; Ferrari *et al*., 2015); Antarctic dragonfish (*Gymnodraco acuticeps*; Flynn *et al*., 2015); humpbacked conch (Lefevre *et al*., 2015); common slipper shell (*Crepidula fornicate*; Noisette *et al*., 2015, 2016); European lobster (*Homarus gammarus*; Small *et al*., 2015); Norway lobster (*Nephrops norwegicus*; Wood *et al*., 2015); and cone-shaped Nassa (*Nassarius conoidalis*; Zhang *et al*., 2015)] or even caused a reduction [e.g. peanut worm (*Sipunculus nudus*; Pörtner *et al*., 1998); velvet swimming crab (*Necora puber*; Small *et al*., 2010); grooved carpet shell (*Ruditapes decussatus*; Fernández-Reiriz *et al*., 2011); Chilean blue mussel (*Mytilus chilensis*; Navarro *et al*., 2013); spiny chromis damselfish (Rummer *et al.*, 2013); common dolphinfish (*Coryphaena hippurus*; Pimentel *et al*., 2014); European squid (*Loligo vulgaris*; Rosa *et al*., 2014b); and hard-shelled mussel (*Mytilus coruscus*; Wang *et al.*, 2015)].

A decreased $\dot{M}O_{2\mathrm{rest}}$ could be interpreted as something positive because, all else being equal, it would imply reduced maintenance costs and potentially higher AAS. The mechanism behind a CO_2_-induced reduction in $\dot{M}O_{2\mathrm{rest}}$ is rarely discussed, but has nonetheless often been interpreted as something negative. It has been suggested that ‘uncompensated extracellular pH might be the trigger for these reductions’ [quote from Stumpp *et al.* (2011); based on Langenbuch and Pörtner (2002) and Pörtner *et al.* (2005)]. Melatunan *et al.* (2011) observed a reduction in $\dot{M}O_{2\mathrm{rest}}$ in common periwinkle that appeared to be compensated for by an increase in anaerobic metabolism, which is not a sustainable strategy and therefore likely to be negative for fitness or even survival, but the mechanism behind a CO_2_-induced reduction in respiratory capacity was not discussed. In embryos of common cuttlefish (*Sepia officinalis*), the observed reduction in $\dot{M}O_{2\mathrm{rest}}$ was interpreted as a form of adaptive short-term metabolic depression invoked to conserve energy (Rosa *et al.*, 2013). Carbon dioxide has also previously been linked to metabolic depression, although this may apply to very high CO_2_ levels, where it has anaesthetic effects (e.g. Guppy and Withers, 1999).

The absolute value of $\dot{M}O_{2\mathrm{rest}}$, and the way it is influenced by one stressor in a given situation, is not necessarily straightforward to interpret. Factors that could contribute to variation in the response are the duration of the CO_2_ exposure, *P*CO_2_ level and temperature. Firstly, one can expect that the longer the animals are exposed to a certain condition, the likelier they are to have compensated for the challenge through acid–base regulation; general stress responses should have subsided, and the harder it might be to detect a remaining effect. In contrast to this reasoning, the few studies using an exposure time of less than a day found no effect of elevated CO_2_ (Rosa and Seibel, 2008; Rivest and Hofmann, 2014), whereas the outcome of long-term experiments (more than a months to a year) were reductions (Fernández-Reiriz *et al*., 2011; Rosa *et al.*, 2014a), no effect (Melzner *et al.*, 2009; Dickinson *et al*., 2013; Gräns *et al.*, 2014; Hildebrandt *et al*., 2014; Uthicke *et al*., 2014; Cunningham *et al.*, 2015; Noisette *et al.*, 2016; Strahl *et al*., 2015; Thor and Dupont, 2015) or increases (Beniash *et al*., 2010; Enzor *et al.*, 2013; Matoo *et al.*, 2013) in $\dot{M}O_{2\mathrm{rest}}$. Overall, it was not possible to detect a direct effect of the exposure times used, of which most were 4 days or more.

Secondly, it could be expected that higher CO_2_ levels led to larger increases in $\dot{M}O_{2\mathrm{rest}}$, because the loading stress would be higher. Curiously, of the few studies that used a high CO_2_ level (5000–33 000 µatm), most found no change in $\dot{M}O_{2\mathrm{rest}}$ (Deigweiher *et al*., 2008; Melzner *et al.*, 2009; Dissanayake and Ishimatsu, 2011; Hammer *et al*., 2011; Tirsgaard *et al.*, 2015b), whereas others found a decrease (Christensen and Colacino, 2000; Small *et al*., 2010; Mingliang *et al*., 2011; Hu *et al*., 2014; Sun *et al*., 2016). Other than that, most of the recent studies have been concerned with climate change, and a relatively narrow range of CO_2_ levels have been used, around 800–1200 µatm, because this is the current RCP8.5 predicted level for the year 2100 (Meinshausen, *et al.*, 2011; Doney, *et al.*, 2012). As these levels are low in a classic physiological sense and cover a narrow range, it is not surprising that they do not explain the wide variation observed in $\dot{M}O_{2\mathrm{rest}}$ response ratios.

Lastly, temperature obviously has a strong influence on physiological performance, and it could be argued that animals at higher temperatures with higher metabolic demands may be more susceptible to possible stressors, such as CO_2_. Unlike the uniform range of CO_2_ treatment levels, animals from a wide range of temperatures have been studied, making it more likely to detect a relationship between temperature and the response to elevated CO_2_, if one existed. But again, negative, neutral and positive effects were spread evenly across the temperature range. Notably, only one of the experiments conducted at the highest temperatures (25–30°C) showed a significant increase in $\dot{M}O_{2\mathrm{rest}}$ (Munday *et al*., 2009), whereas three studies showed a decrease with CO_2_ treatment (Rummer *et al.*, 2013; Rosa *et al.*, 2014b; Wang *et al.*, 2015).

But still, it cannot be ruled out that some of the studies, which measured routine rather than resting $\dot{M}O_{2}$, failed to detect a difference because a truly resting state was not obtained. A truly resting state may, however, be difficult to confirm in some types of animals, such as corals and bivalves. Importantly, the direction (decrease vs. increase) of responses was not affected by overall methodology, indicating that the divergence in the CO_2_ effects, when these are found, may in fact be a result of different physiological mechanisms. A more obvious difference between studies is, of course, that they have been conducted on different species, and identifying the mechanistic background for each of the outcomes will require further and more detailed studies on those particular species. Only a few species have been investigated multiple times but in slightly different experimental conditions. In Atlantic oysters, Beniash *et al*. (2010) found an increase in $\dot{M}O_{2\mathrm{rest}}$ at elevated CO_2_ in juveniles, but no effect in adults, as did Matoo *et al*. (2013). In the purple sea urchin (*Strongylocentrotus purpuratus*), Stumpp *et al*. (2011) found an increase after 21 days, whereas both Stumpp *et al*. (2011) and Padilla-Gamiño *et al*. (2013) found no effect after 4 days. In the Chilean abalone (*Concholepas concholepas*), Manríquez *et al*. (2013) found no effect in adults, whereas Lardies *et al*. (2014) found an increase in $\dot{M}O_{2\mathrm{rest}}$ in juveniles, although with a higher *P*CO_2_ and temperature and shorter treatment duration. In adult blue mussels, Zittier *et al*. (2015) and Sun *et al*. (2016) found no effect of elevated CO_2_ at 15°C, whereas Thomsen and Melzner (2010) found an increase in $\dot{M}O_{2\mathrm{rest}}$ at 8°C. Lastly, in the king scallop (*Pecten maximus*) an increase in $\dot{M}O_{2\mathrm{rest}}$ was found after 50 days (Schalkhausser *et al*., 2014), but not after 30 days, of high-CO_2_ treatment (Schalkhausser *et al*., 2013). There may even be differences in the response between different populations of a species, because, for example, Thor and Oliva (2015) found $\dot{M}O_{2\mathrm{rest}}$ to be elevated after high-CO_2_ exposure in the copepod *Pseudocalanus acuspes* from Skagerrak, whereas the same treatment did not have an effect on individuals from Svalbard.

### Does elevated CO_2_ in general reduce ’aerobic scope?

The variability detected in the response in $\dot{M}O_{2\mathrm{rest}}$ to elevated CO_2_ is also reflected in AAS, which decreases in some studies [jumbo squid (Rosa and Seibel, 2008); yellowstriped and fourline cardinalfish (Munday *et al*., 2009); and Shiba shrimp (Dissanayake and Ishimatsu, 2011)], but not in others [ambon damsel (*Pomacentrus amboinensis*), lemon damsel, brown dottyback (*Pseudochromis fuscus*; Couturier *et al*., 2013); European sea bass (Pope *et al*. 2014); red drum (Esbaugh *et al*., 2016); small-spotted catshark (Green and Jutfelt, 2014); and humpbacked conch (Lefevre *et al*., 2015)], and in fact, even increases in a third, albeit small, group [spiny chromis damselfish (Rummer *et al*., 2013); and Atlantic halibut (Gräns *et al*., 2014)]. Overall, these outcomes were not influenced by methodology, because the proportion of studies finding an effect vs. no effect, and an increase vs. a decrease, was the same for studies measuring $\dot{M}O_{2\max}$ during exercise or after chasing. The relationship between the effect of elevated CO_2_ on $\dot{M}O_{2\mathrm{rest}}$ and the effect of elevated CO_2_ on AAS was weak, which does not support the hypothesis that it is an increase in $\dot{M}O_{2\mathrm{rest}}$ that causes AAS to decrease. Why, therefore, is AAS sometimes affected by CO_2_? Obviously, if it is not because $\dot{M}O_{2\mathrm{rest}}$ changes, it must be $\dot{M}O_{2\max}$ that changes. It has been hypothesized that CO_2_ acts as a limiting stressor, reducing $\dot{M}O_{2\max}$ by, for example, interfering with respiratory pigments [Heuer and Grosell (2014) cites Pörtner *et al.* (2004b), who cites Tamburrini *et al.* (1998)], but no further studies appear to have examined this hypothesis. For the cases of CO_2_-induced reductions of $\dot{M}O_{2\max}$, it has been suggested that it is changes in metabolic pathways, specifically a shift in the balance between anaerobic and aerobic pathways (as reported by Michaelidis *et al.*, 2007), that supress AAS and/or $\dot{M}O_{2\max}$ (Munday, *et al.*, 2009). Schalkhausser *et al.* (2013) found that $\dot{M}O_{2\max}$ and AAS were reduced in the king scallop, but these findings were not replicated in a later study (Schalkhausser *et al.*, 2014), and the authors did not attempt to explain either the decreased $\dot{M}O_{2\max}$ or the discrepancy between their experiments. In the cases where elevated CO_2_ had no effect, this was attributed to a sufficient capacity for acid–base regulation (Melzner, *et al.*, 2009), also with long-term exposure. Maybe for good reasons, i.e. to avoid unfounded speculation, some studies reflect little or only vaguely upon the mechanistic cause for the observed response, whether it was a reduction (e.g. Rosa and Seibel, 2008; Dissanayake and Ishimatsu, 2011; Tirsgaard *et al.*, 2015b) or no change (Schalkhausser *et al.*, 2013; Lefevre *et al.*, 2015). Given the hypothesis that CO_2_ acts as a limiting factor, it is counterintuitive to find that $\dot{M}O_{2\max}$ increases in some animals exposed to elevated CO_2_ . In the Atlantic halibut, increased $\dot{M}O_{2\max}$ appeared to be associated with increased maximal pumping ability of the heart (Gräns, *et al.*, 2014), although it is unclear why CO_2_ would have that effect. Rummer *et al.* (2013) suggested that increased respiratory surface area contributed to an increased $\dot{M}O_{2\max}$, and even hypothesized that an interaction between acidosis and stress could have led to catecholamine release, which increased oxygen delivery to the muscles by reducing the affinity of haemoglobin for oxygen. Couturier *et al.* (2013) suggested increased swimming speed as a possible explanation for increased $\dot{M}O_{2\max}$ when measured in a swim respirometer, which in turn was hypothesized to be associated with behavioural alterations caused by high CO_2_. In other words, it could be that neural effects of exposure to elevated CO_2_ either increase the drive to swim fast or take away any behavioural inhibition that may suppress maximal exercise efforts. In addition, Couturier *et al.* (2013) pointed towards the interesting possibility that elevated CO_2_ does indeed incur energetic costs, which are not measurable in resting conditions, but become evident when the fish are pushed to the limit of their capacity, forcing them to increase $\dot{M}O_{2\max}$. If this suggestion is correct, then an increased $\dot{M}O_{2\max}$ or AAS should not necessarily be interpreted as something promoting survival and fitness, a notion also put forward by Di Santo (2015), although the latter study did not measure $\dot{M}O_{2\max}$*per se*.

As a result of the difficulty (or reluctance) of publishing negative results, it may even be that counting the number of studies showing positive or negative effects of elevated CO_2_ on aerobic performance variables gives an overestimation of the general effects that expected future CO_2_ may have on marine animal respiration and metabolism. One may make the general reflection that the degree of ocean acidification caused by a projected future *P*CO_2_ of ∼1000 µatm does not make the ocean acidic (i.e. pH < 7.0) but will reduce pH from today’s pH ∼8.2 to ∼7.8. At least with regard to teleost fish, this will bring the water pH closer to the blood pH, and if there is any energetic cost involved in maintaining a pH gradient between blood and water, then this cost would be reduced. One may also reflect upon the fact that a pH of 7.8 would be readily tolerated by virtually all freshwater fish, because this is a very ‘average’ freshwater pH. Thus, effective mechanisms needed to handle variations in water pH do exist in teleost fish, although it could be argued that they may have been lost in many marine teleosts living in stable pH conditions. Consequently, there are no obvious reasons to expect that the acidification as such would pose a serious problem, at least for fish, and this may be part of the explanation for the divergent results.

Calcifying invertebrates could be another matter, although results vary even within this group. Although one might have expected $\dot{M}O_{2\mathrm{rest}}$ to be increased more generally by elevated CO_2_ in calcifying organisms, the few existing studies on adult tropical coral do not show an effect of CO_2_ on $\dot{M}O_{2}$ (dark respiration; Takahashi and Kurihara, 2013; Strahl *et al*., 2015; Comeau *et al*., 2016), whereas $\dot{M}O_{2}$ was reduced in a cold-water coral (Hennige *et al*., 2014). Likewise, studies on coral larvae have not found an effect of CO_2_ on $\dot{M}O_{2}$ (Nakamura *et al*., 2011; Putnam *et al*., 2012; Cumbo *et al*., 2013; Edmunds *et al*., 2013; Rivest and Hofmann, 2014). However, the elevation of CO_2_ in itself can still be expected to be problematic for growth and calcification of calcifying organisms (e.g. Fabry, 2008; Ries *et al*., 2009; Gutowska *et al*., 2010; Mingliang *et al*., 2011; Bramanti *et al*., 2013; Kaplan *et al*., 2013; Wolfe *et al*., 2013; Byrne *et al*., 2014; Watson, 2015) and also for fish (e.g. Baumann *et al*., 2012; Frommel *et al*., 2012; Chambers *et al*., 2014) on the background of the increasing number of studies revealing neural effects of relatively modest rises in *P*CO_2_ (see Beyond aerobic scope: behavioural effects of CO_2_).

### Combined effects of temperature and CO_2_ on resting aerobic metabolism

As discussed in Aerobic scope curves across species and time frames, the expectation that elevated temperature causes $\dot{M}O_{2\mathrm{rest}}$ to increase, the magnitude of which will be dependent on the acclimatory ability, is well founded from decades of research. It is much less so for CO_2_, and data seem to reflect this. Putting the two together, which is inevitable from a global change perspective, does not make matters clearer. A prediction could be that CO_2_ is a stressor, which causes an increase in $\dot{M}O_{2\mathrm{rest}}$ that simply adds on top of the increase caused by temperature. Indeed, the combined effect of elevated temperature and CO_2_ on respiration seems to be additive, when looking at it quantitatively. Notably, in several cases the combined outcome is additive simply because there was no measurable effect of CO_2_ on $\dot{M}O_{2\mathrm{rest}}$ (Comeau *et al*., 2010; Dissanayake and Ishimatsu, 2011; Strobel *et al.*, 2012; Arnberg *et al.*, 2013; Padilla-Gamiño *et al.*, 2013; Carey *et al.*, 2014; Rivest and Hofmann, 2014; Schalkhausser *et al*., 2014; Cunningham *et al.*, 2015; Lefevre *et al.*, 2015; Noisette *et al.*, 2016; Small *et al.*, 2015; Zhang *et al.*, 2015), whereas in other cases either temperature or CO_2_ alone had an effect (Rosa and Seibel, 2008; Munday *et al.*, 2009; McElroy *et al*., 2012; Matoo *et al.*, 2013; Hildebrandt *et al*., 2014; Flynn *et al.*, 2015; Kreiss *et al.*, 2015). There are only a few cases suggesting synergistic effects, where the change during combined exposure is significantly higher than expected from the sum of the isolated effects, one example being a coral reef cardinalfish (fourline cardinalfish; Munday *et al.*, 2009), the other being an Antarctic fish (bald notothen; Enzor, *et al.*, 2013). The mechanism behind these synergistic effects, however, is not clear. It is also worth noting that several of the studies failing to show a significant interaction fall below the additive line (Fig. 10A and C). That is, in many cases temperature causes an increase in $\dot{M}O_{2\mathrm{rest}}$ as expected, but when the two environmental stressors are applied together, the increase is significantly lower than expected from an additive response [purple sea urchin (Catarino *et al.*, 2012); dwarf cushion star (McElroy *et al.*, 2012); cauliflower coral (Cumbo *et al*., 2013); striped rockcod, dusky rockcod (*Trematomus newnesi*; Enzor *et al.*, 2013); *Amphiura filiformis* (Carey *et al.*, 2014); and white-spotted bamboo shark (Rosa *et al.*, 2014a)].

Obviously, there can be arguments both for and against an increase in $\dot{M}O_{2\mathrm{rest}}$ being negative for survival or fitness (depending, for example, on nutritional status and food availability), but if one assumes that saving energy is an adaptive strategy for the average animal, an antagonistic interaction can be interpreted such that CO_2_ alleviates some of the costs associated with increased temperature. In contrast, in the few instances where temperature causes a decrease in $\dot{M}O_{2\mathrm{rest}}$, CO_2_ seems to have the opposite effect and causes an increase in $\dot{M}O_{2\mathrm{rest}}$ despite the expected decrease from temperature alone [sea urcin (*Echinometra* sp.; Uthicke *et al.*, 2014); and Atlantic cod (Kreiss *et al.*, 2015)] or at least causing the decrease to be smaller than expected [common periwinkle (Melatunan *et al.*, 2011); and hard-shelled mussel (Wang *et al.*, 2015)], which is probably not beneficial (again, if it is assumed that low $\dot{M}O_{2\mathrm{rest}}$ is beneficial). A decrease in $\dot{M}O_{2\mathrm{rest}}$ with increased temperature is clearly not the expected outcome, and it is noteworthy that it is observed only in invertebrates, whereas the majority of the teleosts investigated show no change or the expected increase in $\dot{M}O_{2\mathrm{rest}}$.

### Combined effects of temperature and CO_2_ on absolute aerobic scope

The predictions from the OCLTT hypothesis for how elevated temperature and CO_2_ in combination affect AAS are relatively straightforward (Fig. 1C and D). Two scenarios are commonly depicted; one where interaction between CO_2_ and temperature is additive (CO_2_ reduces AAS over the entire temperature range), and one where it is synergistic (CO_2_ narrows the thermal windows, by causing a further reduction in AAS with increased or decreased temperature). Synergistic effects will only be worse than additive effects in a global warming scenario if it is assumed that the animal is presently living near *T*_optAAS_ (which most animals will strive to do, according to the OCLTT). Although there is currently a limited number of studies that have looked at the effect of both temperature and CO_2_ on AAS, the data seem to support an additive outcome [jumbo squid (Rosa and Seibel, 2008); and European sea bass (Pope *et al.*, 2014)], of which some are simply a result of a very small or absent effect of CO_2_ [Atlantic halibut (Gräns *et al.*, 2014); and humpbacked conch (Lefevre *et al.*, 2015)]. In a few instances, a positive antagonistic effect is evident, where temperature seems to alleviate the negative effect of CO_2_ [Shiba shrimp (Dissanayake and Ishimatsu, 2011); and king scallop (Schalkhausser, *et al.*, 2014)]. Interestingly, the nature of the interaction between CO_2_ and temperature can vary within a species, as for some coral reef fish, where it can be antagonistic with a moderate temperature increase and turn additive at higher temperature (yellowstriped and fourline cardinalfish; Munday *et al.*, 2009). Furthermore, there seems to be a correlation between the response and either life stage or type of fish, because the cases with negative combined effects (Fig. 10C) were adult coral reef fish, whereas the unaffected fish were juvenile temperate fish, making it difficult to speculate on the underlying factors. Obviously, more studies of this kind on a wider range of fish species are needed to draw firm conclusions, but at least it is clear that synergistic effects have not been observed. For the animal, it is perhaps less important if it is succumbing to an additive or synergistic effect. However, for scientists trying to predict ecological effects of global change, an important implication of an additive rather than synergistic effect (when CO_2_ has an effect at all) is that it is inherently easier to incorporate additive effects in mathematical models.

### Beyond aerobic scope: behavioural ’effects of CO_2_

Aerobic scope is a conceptually attractive measure of performance, because in theory it represents the overall capacity, that is, the amount of energy that can be devoted to different activities at any given point. This capacity is nonetheless still theoretical, because it is difficult to know how and when an animal uses this capacity in nature, where many other factors are at play (e.g. food availability and conspecifics; Farrell, 2013, 2016). A recent meta-analysis on reproduction and survival concluded synergistic effects to be dominant (Harvey *et al*., 2013), which contrasts with the dominantly additive effect on respiratory performance found in the present analysis, indicating that the underlying mechanisms may differ.

When it comes to the effect of climate change on variables beyond AAS, there is an additional twist to the story, namely behaviour. So far, behavioural studies on the interaction between warming and acidification are limited to a recent study by Ferrari *et al.* (2015) on coral reef fish [ambon damsel and Nagasaki damsel (*Pomacentrus nagasakiensis*)]. Strikingly, they found the interactive effect on predation rate to be synergistic (i.e. there was no effect of CO_2_ or temperature by themselves, but there was an effect when the two were combined), whereas prey selectivity was antagonistically affected (i.e. both temperature and CO_2_ by themselves reversed prey preference, and any preference was completely abolished when both stressors were combined). Behavioural effects of CO_2_ or low pH were reviewed in detail by Briffa *et al.* (2012) and more recently by Clements and Hunt (2015) and Nagelkerken and Munday (2016), who found that the majority of published studies find an effect of elevated CO_2_. However, these direct effects of CO_2_ on behaviour are not part of the OCLTT framework and were judged to have ‘low confidence’ in the most recent edition of the IPCC report (Pörtner *et al*., 2014).

For some species, it is not surprising that CO_2_ does not have an effect. The epaulette shark (*Hemiscyllium ocellatum*), which is a shallow-reef species, is exposed to rather severe hypoxia and hypercapnia on a diurnal basis, and in addition to being hypoxia tolerant (e.g. Speers-Roesch *et al*., 2012a,b) it is therefore likely to be tolerant of elevated CO_2_, and accordingly, it is not behaviourally affected (Heinrich *et al.*, 2014, 2016). This may also be the case for Atlantic cod, which is known to feed in hypoxic–hypercapnic waters (Strand and Huse, 2007; Neuenfeldt *et al.*, 2009) and which is also not behaviourally affected by elevated CO_2_ (Jutfelt and Hedgärde, 2015). The behavioural changes seen in other cases can easily be interpreted as negative, such as being attracted to predators, the wrong habitat or the wrong food (e.g. Vargas *et al.*, 2013; Munday *et al.*, 2014; Sundin and Jutfelt, 2016), failing to learn (Chivers *et al*., 2014), altered prey handling (Dodd *et al*., 2015) and loss of the ability to respond to important cues (e.g. Dixson *et al*., 2010; Simpson *et al*., 2011; Lönnstedt *et al.*, 2013; Munday *et al*., 2013; Allan *et al.*, 2014; Manríquez *et al*., 2013, 2014; Watson *et al*., 2014; Sui *et al*., 2015). The interpretation can be more difficult when it comes to something like lateralization (e.g. Jutfelt *et al*., 2013) or activity (e.g. Pimentel *et al*., 2014; Green and Jutfelt, 2014; Spady *et al*., 2014). Although a ‘hard-wired’ preference for left or right might decrease response time, it can also be argued that it limits the options and makes an individual predictable. The benefit of being more or less active depends on the situation; if an animal is more active it might be more likely to find food or conspecifics, but it might also be more visible and thereby vulnerable to predation. Importantly, in the present context, behavioural changes related to elevated *P*CO_2_ could affect measurements of $\dot{M}O_{2\mathrm{rest}}$ and $\dot{M}O_{2\max}$ and could explain the elevation of these variables seen in some studies.

In contrast to the limited understanding of whether and how elevated *P*CO_2_ affects $\dot{M}O_{2\mathrm{rest}}$ and AAS, there is now an increasing understanding of the mechanism that underlies the behavioural alterations. Since first suggested from experiments showing that the effects of CO_2_ on olfaction and lateralization can be reversed by treatment with the GABA_A_ receptor antagonist gabazine (Nilsson *et al.*, 2012), several studies have linked these clearly neurological changes to an altered function of this major inhibitory neurotransmitter receptor. Thus, Chivers *et al.* (2014) showed that high-CO_2_-induced learning deficiency in ambon damselfish could be reversed by gabazine treatment, and the direct neuronal effect of continuous CO_2_ exposure on retinal function in spiny chromis damselfish was also reversed by gabazine (Chung *et al.*, 2014). Subsequently, similar results have been seen in temperate marine teleosts [three-spined stickleback (*Gasterosteus aculeatus*; Lai *et al*., 2015); and split-nose rockfish (*Sebastes diploproa*; Hamilton *et al.*, 2014)], and maybe more surprisingly, in the early freshwater life stage of pink salmon (Ou *et al.*, 2015) and the facultative air-breathing striped catfish (*Pangasianodon hypophthalmus*; Regan *et al*., 2016). Indeed, the effects of CO_2_ on the GABA_A_ receptor are apparently not limited to fish, because they have also been found in a gastropod, the humpbacked conch (Watson *et al.*, 2014). The link between elevated CO_2_ and the GABA_A_ receptor probably lies in the fact that this receptor is an ion channel with conductivity for Cl^−^ and HCO_3_^−^, and disruption of the neuronal gradients for these ions could readily alter its function (Nilsson *et al*., 2012; Heuer and Grosell, 2014). For fish, acid–base regulation is very much dependent on regulation of the levels of Cl^−^ and HCO_3_^−^, and it is perhaps not so surprising that neural functions depending on these ions could be affected by hypercapnia.

Over the last few years, it has thus become clear that neural functions of many marine species are vulnerable to ocean acidification. Although reduced AAS may reduce fitness in the long run, it can be acutely detrimental for an animal not to respond with an appropriate behaviour to cues in its environment. Likewise, it does not matter how large and unaffected AAS is by ocean acidification, if behaviour becomes severely maladaptive. As an example, a study on humpbacked conch showed that AAS *per se* was not impaired by elevated CO_2_, whereas it had a negative impact on behaviour, so that a larger proportion of snails failed to elicit an escape response when exposed to odour from a predator (Watson *et al*., 2014). In this case, one would arrive at two completely different predictions for the future of these snails, had only one of the variables been investigated. It is therefore crucial to implement both physiology and animal behaviour, and the consequences of alterations caused by warming and acidification, in attempts to predict the impact of climate change (Nagelkerken and Munday, 2016).

From an experimental point of view, care has also to be taken to assure that measurements of $\dot{M}O_{2\mathrm{rest}}$ and $\dot{M}O_{2\max}$, in ocean acidification conditions, are not confounded by behavioural alterations, including changes in activity or drive to exercise. Even more worryingly, although studies have shown negative effects of temperature and CO_2_ on oxygen uptake to be alleviated through developmental or transgeneration acclimation (Donelson *et al.*, 2011, 2012; Donelson and Munday, 2012; Donelson, 2015), this may not be the case for behavioural abnormalities induced by elevated CO_2_ (Allan *et al.*, 2014; Munday, *et al.*, 2014; Welch *et al*., 2014). Interestingly, a recent study on three-spined stickleback showed that offspring from parents exposed to high CO_2_ had lower survival when reared in control conditions compared with offspring reared in high-CO_2_ water (Schade *et al.*, 2014), but the effect appeared to be opposite for growth. Obviously, more studies are warranted to characterize these seemingly complex relationships.

### Conclusions

Bozinovic and Pörtner (2015) argue that ‘Development of unifying concepts such as OCLTT is relevant for interpreting existing and future findings in a coherent way and for projecting the future ecological and evolutionary effects of climate change on whole-organism and ecosystem functioning’. In one sense, it is easy to agree with this statement, because the creation of predictive and testable mechanistic models can be hugely successful in inspiring researchers and moving science forward. Indeed, the idealized thermal performance curves proposed under the concept of OCLTT for AAS and the hypothesized influence of CO_2_ on these curves are widely used as a framework for research. It is also a healthy sign that models are questioned, tested, rejected or improved, in light of available and new data. The patterns emerging from the present analysis indicate that such performance curves may not fit the majority of marine animals, particularly for the combined effects of high temperature and high CO_2_. Even within one species, the effect of elevated CO_2_ and temperature, and thereby the predicted outcome of climate change, may vary, depending on which variable is examined, and the effect on one variable may not predict the effect on another (e.g. Gräns *et al*., 2014; Watson *et al*., 2014).

The idea that ‘Available knowledge suggests unifying physiological principles of CO_2_ effects, across animal groups and phyla’ (Pörtner, 2010) is not supported by the data reviewed here. Synergistic effects of ocean acidification on variables such as $\dot{M}O_{2\mathrm{rest}}$ and AAS appear to be rare; instead, additive effects seem to prevail, with many examples also of antagonistic effects. This variability indicates intricate underlying mechanisms. Although it is clear that climate change can have severe effects and that AAS might be a useful parameter for modelling the outcomes in some cases, species-by-species research and attempts to uncover the cause of different responses are still essential, and generalizations should be made with caution. The recent discovery of neurological and thereby behavioural effects of CO_2_ exposure is currently treated as a parallel threat to marine ectotherms and a parallel area of research, because it is not linked to the OCLTT framework. However, the neurological alterations, although equally important on their own, may also prove to be confounding factors in experimental studies of other aspects of physiological performance, including resting and maximal rates of oxygen uptake.

## Supplementary material

Supplementary material is available at *Conservation Physiology* online.

## Funding

This work was supported by the Research Council of Norway, the University of Oslo, and the European Cooperation in Science and Technology (COST) action ‘Conservation Physiology of Marine Fishes’ [FA1004].

## Supplementary Material

Supplementary Data

Supplementary Data
