# Supplementary material for: Are global warming and ocean acidification conspiring against marine ectotherms? A meta-analysis of the respiratory effects of elevated temperature, high CO2 and their interaction
Source: Conserv Physiol. 2016 Mar 23;4(1):cow009. doi: 10.1093/conphys/cow009 (PMC4922249; doi:10.1093/conphys/cow009)
Supplement: Supplementary Data [file supp_4_1_cow009__index.html]

Supplementary Data 

# Are global warming and ocean acidification conspiring against marine ectotherms? A meta-analysis of the respiratory effects of elevated temperature, high CO2 and their interaction

## Supplementary Data

Supplementary Data

- Supplementary Data - Supplementary Data
